# Mitochondrial COI gene is valid to delimitate Tylenchidae (Nematoda: Tylenchomorpha) species

**DOI:** 10.21307/jofnem-2020-038

**Published:** 2020-04-24

**Authors:** Mengxin Bai, Xue Qing, Kaikai Qiao, Xulan Ning, Shun Xiao, Xi Cheng, Guokun Liu

**Affiliations:** 1 Key Laboratory of Biopesticide and Chemical Biology, Ministry of Education, Fujian Agriculture and Forestry University, 350002, Fuzhou, Fujian, China; 2 Department of Plant Pathology, Nanjing Agricultural University, Nanjing, 210095, China; 3 State Key Laboratory of Cotton Biology, Institute of Cotton Research of CAAS, 455000, Anyang, Henan, China

**Keywords:** *Lelenchus leptosoma*, Phylogeny, 28S rRNA, 18S rRNA, Species identification

## Abstract

Tylenchidae is a widely distributed soil-inhabiting nematode family. Regardless their abundance, molecular phylogeny based on rRNA genes is problematic, and the delimitation of taxa in this group remains poorly documented and highly uncertain. Mitochondrial Cytochrome Oxidase I (COI) gene is an important barcoding gene that has been widely used species identifications and phylogenetic analyses. However, currently COI data are only available for one species in Tylenchidae. In present study, we newly obtained 27 COI sequences from 12 species and 26 sequences from rRNA genes. The results suggest that the COI gene is valid to delimitate Tylenchidae species but fails to resolve phylogenetic relationships.

Tylenchidae is a widely distributed soil-inhabiting nematode family characterized by a weak stylet, an undifferentiated non-muscular pharyngeal corpus, and a filiform tail. Currently, it comprises 412 nominal species belongs to 44 genera and estimated species number ranged from 2,000 to 10,000 species (Qing and Bert, 2019). Regardless of their abundance, the delimitation of taxa in this group remains poorly documented and highly uncertain. Consequently, there is no consensus regarding their classification from species level up to family level (Andrássy, 2007; Brzeski, 1998; Qing and Bert, 2019; Siddiqi, 2000).

With the improved availability of genetic sequencing, molecular sequences in species diagnosis and phylogeny analysis have consolidated them as one of the most powerful tools in current taxonomy. Among marker genes, the ribosomal RNA (rRNA) genes are being used as the standard barcode for almost all animals and successfully resolved several groups in Nematoda (Bert et al., 2008; Holterman et al., 2006; Subbotin et al., 2006). However, rRNA genes are problematic in Tylenchidae phylogeny and the unresolved status is unlikely to be improved by intensive species sampling (Qing et al., 2017; Qing and Bert, 2019). Therefore, finding a proper molecular marker gene is crucial for the Tylenchidae study. In this study we examined the mitochondrial Cytochrome Oxidase I gene (COI) of 12 species belong to Tylenchidae (*sensu* (Geraert, 2008)), the goal is to evaluate the potential of COI sequences for the identification of Tylenchidae species; and compare the resolution, sequences variability, and tree topologies obtained from one COI and two rRNA markers (i.e. 18S and the 28S rRNA).

## Materials and methods

### Samples collection and processing

Soil samples were collected in China from 2018 to 2019. The details on sampling locations and habitats were given in Table 1. The nematodes were extracted from soil samples by Baermann tray and subsequently collected by a 400 mesh sieve (37 μm opening) after 24 hr of incubation. For morphological analysis, the extracted nematodes were manually picked up, fixed with 4% formalin, rinsed several times with deionized water and then transferred to anhydrous glycerin, following the protocol of Seinhorst (1962) and Sohlenius and Sandor (1987).

**Table 1.** List species examined in this study and their corresponding sampling locations.

Species	GPS coordinates	Al.	Vegetation environment
*Labrys fujianensis*	26°04´52.9˝N,119°14´26.7˝E	28	Scrubland soil with ferns and bamboo
*Labrys fuzhouensis*	26°08´57.6˝N,119°17´34.4˝E	107	Rhizosphere of *Alpinia zerumbet*
*Coslenchus rafiqi*	26°05´08.2˝N,119°14´10.0˝E	27	Swamp soil
*Coslenchus costatus*	26°05´00.9˝N, 119°14´32.6˝E	25	Rhizosphere soil of bamboo
*Boleodorus thylactus*	26°08´57.6˝N,119°17´34.4˝E	107	Rhizosphere soil of *Alpinia zerumbet*
*Aglenchus geraerti*	26°09´09.2˝N,119°17´35.7˝E	88	Rhizosphere soil of grass near the bamboo
*Basiria aberrans*	26°09´56.3˝N,117°55´34.2˝E	644	Rhizosphere soil of peanut
*Filenchus vulgaris*	26°05´00.9˝N,119°14´32.6˝E	25.	Rhizosphere soil of bamboo
*Lelenchus leptosoma* 1	26°05´00.9˝N,119°14´32.6˝E	25.	Rhizosphere soil of bamboo
*Lelenchus leptosoma* 2	26°08´57.3˝N,119°17´34.1˝E	107	Rhizosphere soil of *Litchi chinensis*
*Malenchus bryanti*	43°48´53.1˝N,125°24´40.3˝E	225	Rhizosphere soil of aspen
*Tylenchus arcuatus*	26°05´23.9˝N,119°14´00.3˝E	12	Rhizosphere soil of locust tree
*Psilenchus hilarulus*	26°05´09.4˝N,119°13´50.2˝E	7	Rhizosphere soil of grass

**Note:** Al, altitude given in m.a.s.l.

### Morphological analysis

Measurements and photography were made from slides using Nikon Eclipse Ni-U 931609 Microscope (Nikon Corporation, Tokyo, Japan). Illustrations were prepared manually based on light microscope drawings and edited with Adobe Illustrator CS3 and Adobe Photoshop CS3.

For scanning electron microscopy (SEM), the samples were fixed by formalin, gradually washed with water and post-fixed with 2% PFA + 2.5% glutaraldehyde in 0.1M Sorensen buffer, then washed and dehydrated in ethanol solutions and subsequently critical point dried with CO_2_. After mounting on stubs, the samples were coated with gold by JFC-1200 and observed with a JSM-3680 (JEOL, Tokyo, Japan).

### Molecular analysis

The fresh nematodes were directly used for DNA extraction. The single nematode was placed in the 10 μl worm lysis buffer (50 mM KCl, 10 mM Tris pH 8.3, 2.5 mM MgCl_2_, 0.45% NP40, 4.5% Tween 20, pH = 8.3) on a glass slide. The nematode cuticle was broken by a needle and subsequently transferred to a 200 μl Eppendorf tube. After 1 min for freezing in liquid nitrogen, 1 μl proteinase K (1.0 mg/ml) was added and incubated for 1 h at 65˚C and 10 min at 95˚C.

The 18S rRNA was amplified with primers 1096F (5´-GGT AAT TCT GGA GCT AAT AC-3´), 988F (5´-CTC AAA GAT TAA GCC ATG C-3´), 1912R (5´-TTT ACG GTC AGA ACT AGG G-3´), 1813F (5´-CTG CGT GAG AGG TGA AAT-3´), and 2646R (5´-GCT ACC TTG TTA CGA CTT TT-3´) (Holterman et al., 2006). The D2-D3 domains of 28S rRNA (28S) were amplified with primers D2A (5´-ACA AGT ACC GTG AGG GAA AGT-3´), D3B (5´-TCG GAA GGA ACC AGC TAC TA-3´) (Nunn, 1992). The cytochrome c oxidase subunit 1 (COI) gene fragment was amplified using JB3 (5´-TTT TTT GGG CAT CCT GAG GTT TAT-3´) and JB4.5 (5´-TAA AGA AAG AAC ATA ATG AAA ATG-3´) (Bowles et al., 1992). The PCR products were sent for sequencing at BioSune Ltd. (Shanghai, China). The newly obtained sequences were deposited in GenBank (accession numbers MN542198-MN542210 for 18S, MN542185-MN542197 for D2-D3 of 28S, MN577595-MN577621 for COI).

The obtained sequences were analyzed with other relevant reference sequences available in the PPNID database (Qing et al., 2020). Multiple alignments of rRNA genes were made using the Q-INS-I algorithm of MAFFT v. 7.205 (Katoh and Standley, 2013) and the COI gene was aligned using TranslatorX (Abascal et al., 2010) under the invertebrate mitochondrial genetic code. The best-fitting substitution model was estimated using AIC in jModelTest v. 2.1.2 (Darriba et al., 2012). Maximum likelihood (ML) and Bayesian inference (BI) was performed at the CIPRES Science Gateway (Miller et al., 2010) using RAxML 8.1.11 (Stamatakis et al., 2008) and MrBayes 3.2.3 (Ronquist et al., 2012), respectively. ML analysis included 1,000 bootstrap (BS) replicates under the GTRCAT model. Bayesian phylogenetic analysis was carried out using the GTR + I + G model, analyses were run for 5 × 10^6^ generations and Markov chains were sampled every 100 generations and 25% of the converged runs were regarded as burn-in. Gaps were treated as missing data for all phylogenetic analysis. ML bootstrap values and posterior probabilities (PP) were plotted on Bayesian 50% majority rule consensus trees using Tree View v. 1.6.6 (Page, 1996) and Illustrator CS3.

## Results

**Figure 1:**
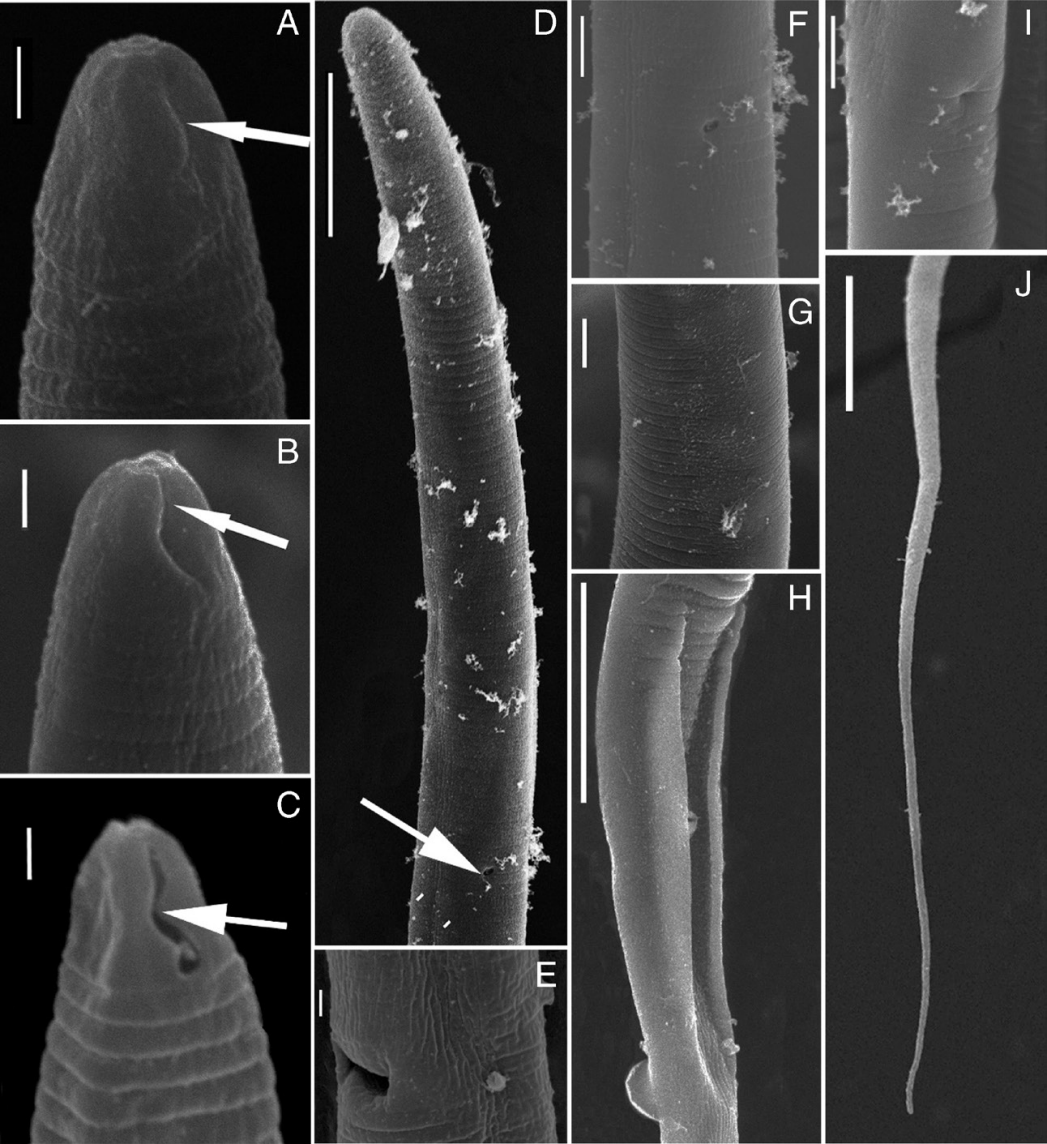
SEM pictures of *Lelenchus leptosome* population 1, 2 (de Man, 1880; Andrássy, 1954). (A, B) *Lelenchus leptosome* population 1; (C-J) *Lelenchus leptosome* population 2. (A, B, C) lip region; (D) anterior body (excretory pore indicated by arrow); (E) lateral view of the vulva; (F) excretory pore; (G) annulation at mid-body; (H) lateral view of cloacal aperture; (I) anus; (J) tail. (Scale bars: A, B, C, E, G=1 μm; D, H, J=10 μm; F, I=2 μm).

**Figure 2:**
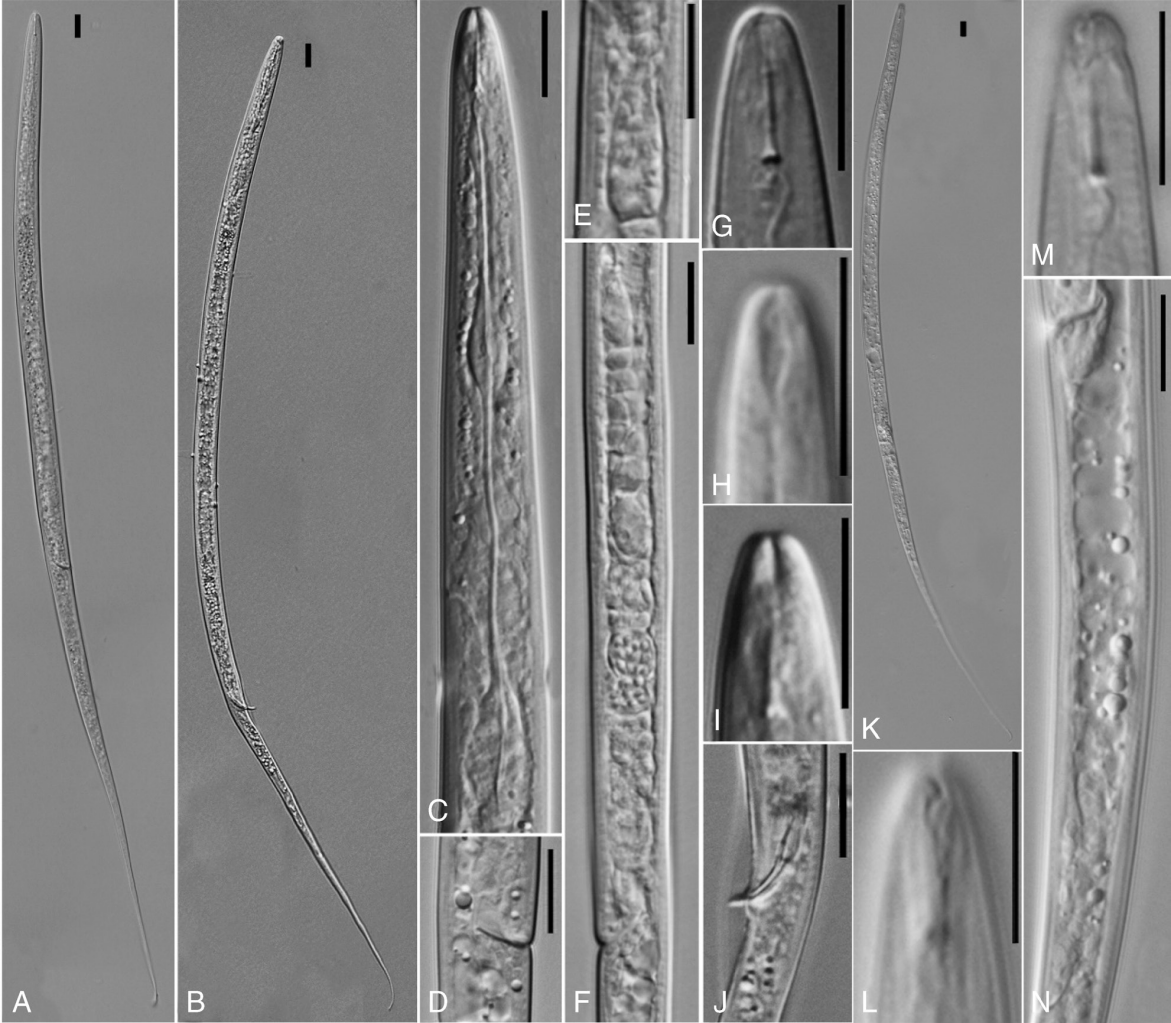
LM pictures of *Lelenchus leptosome* populations 1 and 2. (K-N) *Lelenchus leptosome* population 1; (A-J) *Lelenchus leptosome* population 2. (A, B, K) body habitus; (C) anterior body; (D) ventral view of the vulva; (E) pharyngeal bulb; (F) lateral view of female reproductive system; (G-I, L, M) different image planes of cephalic region; (J) spicule and gubernaculum; (N) vulval to the anus. (Scale bar: 10 μm).

**Figure 3:**
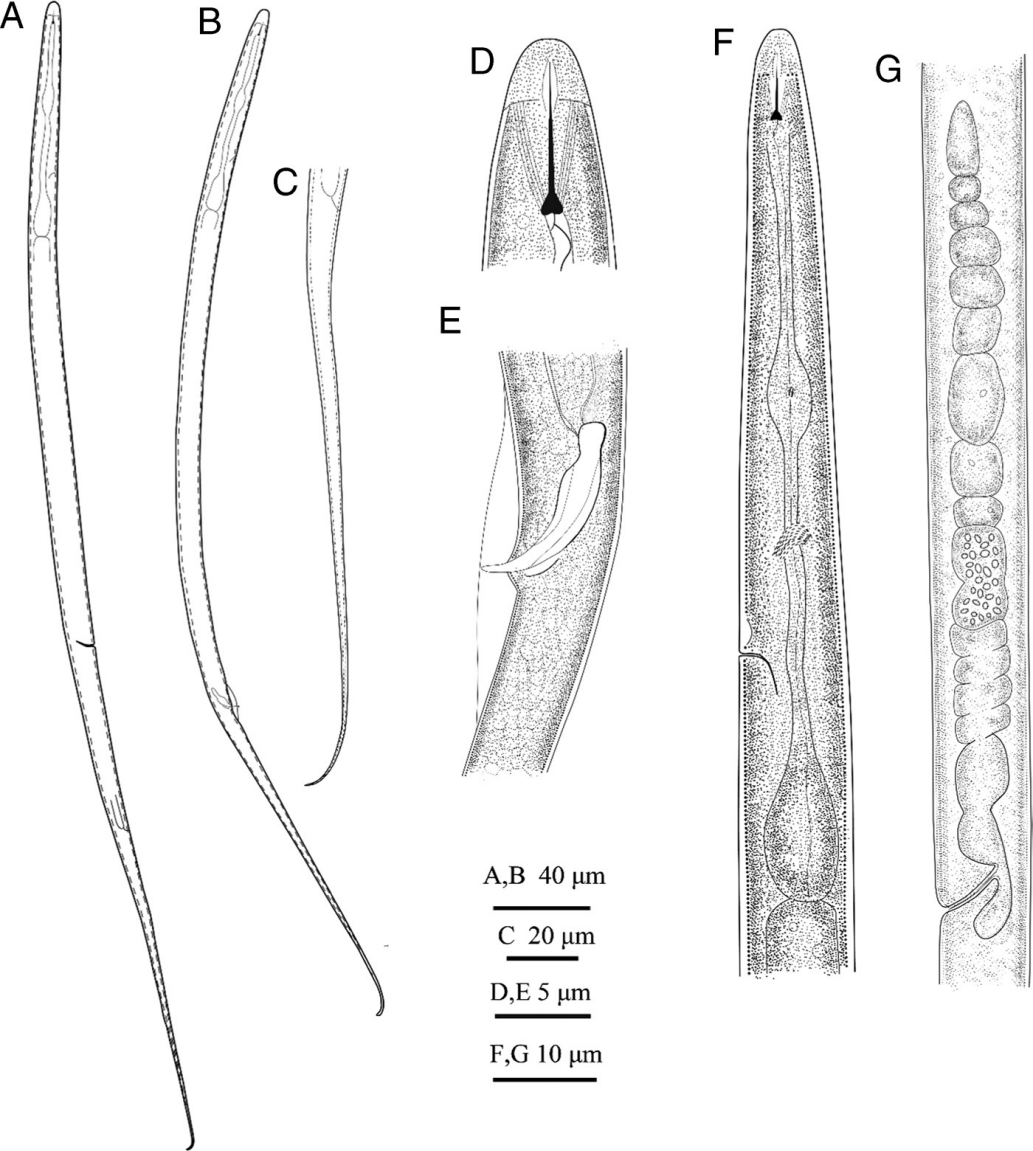
Line drawing of *Lelenchus leptosoma* population 2. (A, C, D, F) female; (B, E) male; (A, B) body habitus; (C) tail; (D) cephalic region; (E) spicule and gubernaculum; (F) anterior body; (G) reproductive system.

To evaluate the validation and robustness of COI phylogeny in comparison to well-established rRNA phylogeny, we newly sequenced corresponding 28S and 18S rRNA of analyzed Tylenchidae species. Our results concur with previous studies that both regions show serious limitations: phylogenies are poorly resolved and support values do not agree with each other (Qing et al., 2017, 2018). In general, the newly sequenced species are placed in the same cluster or closely related to their corresponding species in GenBank (the morphology details are given in Figs. 1-3 and Supplementary Tables 1-4 in https://doi.org/10.6084/m9.figshare.12110667.v1). Exceptionally, two newly recovered *Lelenchus leptosoma* populations (MN542202, MN542203) are placed separately, one population sister to *Lelenchus brevislitus* KU234167 (PP = 1, BS = 97) while another sister to all *Lelenchus* species (PP = 1, BS = 100). The morphological and morphometric comparison showed that two *L. leptosoma* populations were similar (Fig. 2; Supplementary Table 3 in https://doi.org/10.6084/m9.figshare.12110667.v1) with only few differences: e.g. the excretory pore is more anterior in *L. leptosoma* population 2 than population 1 (57.5-73.8 vs 74.4-77.0 μm), and *L. leptosoma* population 2 has shorter pharynx than population 1 (66.0-88.6 vs 89.5-100 μm). Moreover, SEM analysis (Fig. 1) suggested that *L. leptosoma* population 2 has a broader amphidial aperture than population 1. These differences appear in the variation range of *L. leptosoma* stated in the study of Geraert (2008). With the limited knowledge of this genus and overall problematic taxonomy in Tylenchidae, here we considered these differences as intra-specific variations of *L. leptosoma*.

We obtain 27 newly generated COI sequences from 12 species with lengths ranging from 436 bp to 445 bp. The identification of our representatives was confirmed by their key morphological features (Supplementary Figs. 1-15 in https://doi.org/10.6084/m9.figshare.12110667.v1) together with rRNA molecular evidence. We compared compositional bias of COI sequences and the result suggested Tylenchidae has similar GC content to Hoplolaimina (sensu Siddiqi, 2000) in three codon positions but different from Criconematina (sensu Siddiqi, 2000) in GC content of first and third codon position (Table 2). The analysis of genetic distance suggested that most species can be well-separated except for two reciprocally similar genera *Aglenchus* and *Coslenchus* (Table 3).

**Table 2.** The compositional bias (GC content) and 1st, 2nd, and 3rd codon position nucleotide alignments.

	Taxa		
Nucleotide composition	Tylenchidae	Criconematina	Hoplolaimina
GC	28.72	22.54	29.42
GC 1st	39.80	28.07	38.82
GC 2nd	35.14	35.07	36.61
GC 3rd	11.23	4.48	12.84

**Table 3.** The p-distance of COI gene between studied Tylenchidae species.

	LFJ	LFZ	CR	AG	BA	BT	PH	CC	FV	LL1	LL2	MB	TA	LB
LFJ	99.5													
LFZ	78.9	99.5												
CR	83.0	81.4	100											
AG	83.2	81.3	99.8	99.5										
BA	79.8	76.2	81.5	81.3	99.5									
BT	78.7	76.6	82.4	82.3	79.4	99.8								
PH	72.5	73.5	77. 8	77.7	73.2	77.2	100							
CC	80.5	78.2	87.5	87.3	79.8	80.8	75.7	98.6						
FV	82.4	79.5	83.3	83.2	80.9	82.5	76.3	82.4	100					
LL1	76.8	76.8	81.0	81.1	71.7	73.9	70.8	77.6	75.1	96.0				
LL2	79.2	81.2	84.4	84.2	75.5	79.0	75.1	82.8	80.1	86.9	97.7			
MB	81.1	78.8	82.6	82.5	78.1	81.8	74.8	81.0	82.7	74.0	79.6	99.7		
TA	82.4	79.2	88. 9	88.8	81.8	85.6	78.5	83.7	83.8	76.7	81.3	84.7	100	
LB	84.1	81.2	86.6	87.0	81.5	81.5	72.9	87.2	84.3	77.9	84.6	84.0	84.9	0

**Notes:** LFJ, *Labrys fujianensis*; LFZ, *Labrys fuzhouensis*; CR, *Coslenchus rafiqi*; AG, *Aglenchus geraerti*; BA, *Barsiria aberrans*; BT, *Boleodurus thylactus*; PH, *Psilenchus hilarulus*; CC, *Coslenchus costatus*; FV, *Filenchus vulgaris*; LL1, *Lelenchus leptosoma* 1; LL2, *Lelenchus leptosoma*; MB, *Malenchus bryanti*; TA, *Tylenchus arcuatus*; LB, *Lelenchus brevislitus*.

**Figure 4:**
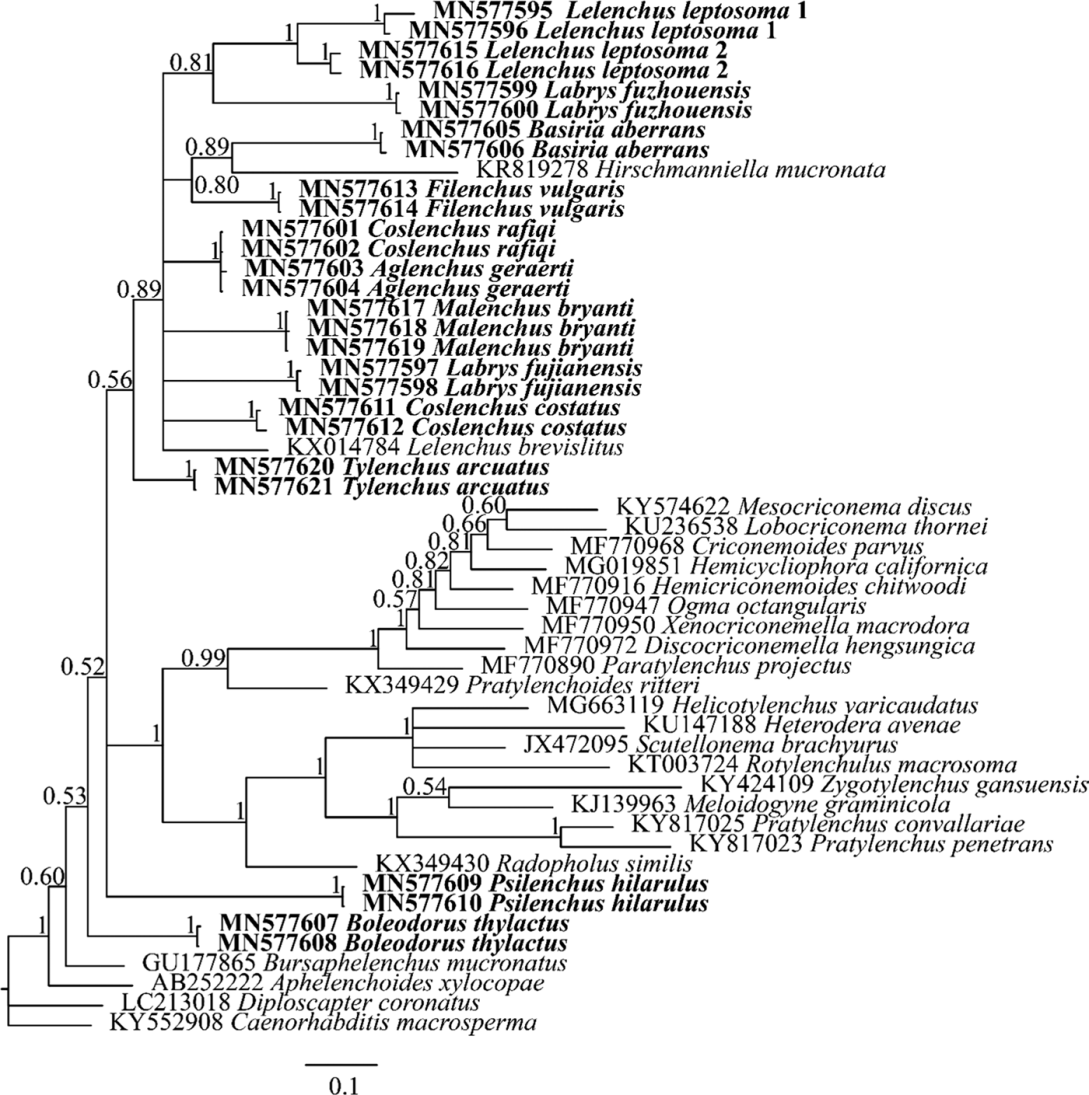
Bayesian 50% majority rule consensus tree interfered with mitochondrial COI gene. New sequences original to this study are indicated in bold. Branch support is PP value in BI analysis.

**Figure 5:**
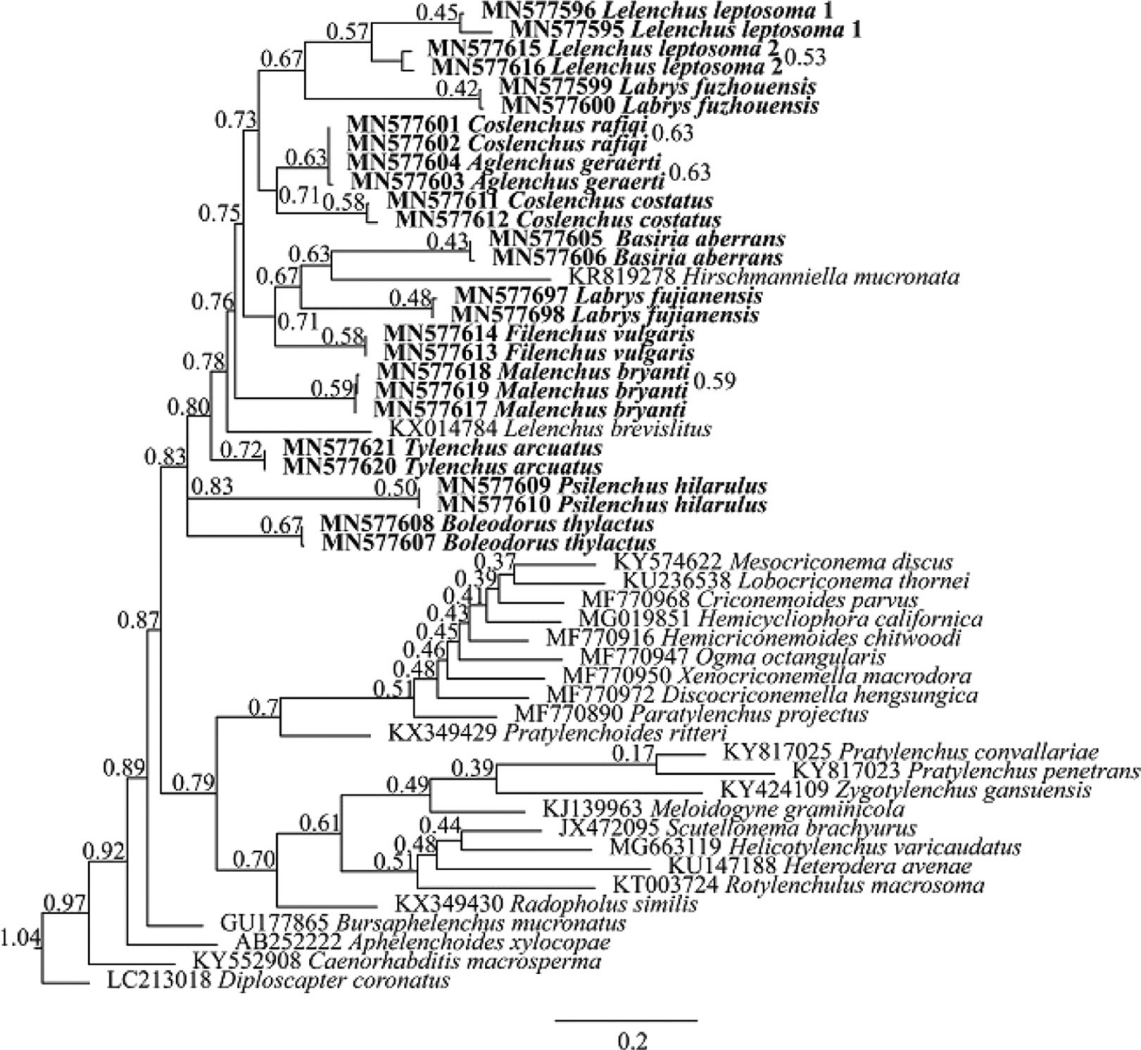
The maximum likelihood tree interfered on the mitochondrial COI gene. New sequences original to this study are indicated in bold. Branch support is BS value from ML analysis.

A total of 52 species in Tylenchomorpha and outgroups (alignment of 1,581 characters) were used for COI phylogeny analysis. The resulting ML and BI trees are largely divergent in topologies, and therefore their phylogenies were presented separately. In both ML and BI analyses, *Hirschmanniella mucronata* (KR819278) was placed as a sister to *Basiria aberrans* (MN577605, MN577606). Such placement was contrary to its morphological assignment and rRNA-based phylogeny (Bert et al., 2008). Since this standalone sequence was not supported by morphology, and other related species (e.g. *Pratylenchus* spp.) were properly placed, we considered likely that this sequence had been mislabeled. On the basis of this assumption, the monophyly of Tylenchidae was moderately supported (BS = 83) by ML analysis but not supported by BI analysis (split into three clusters, Figs. 4, 5). In all analyses, individuals of the same population were clustered together, either in a fully supported clade in BI (PP=1) or weakly supported clade in ML (BS from 43 to 72).

**Figure 6:**
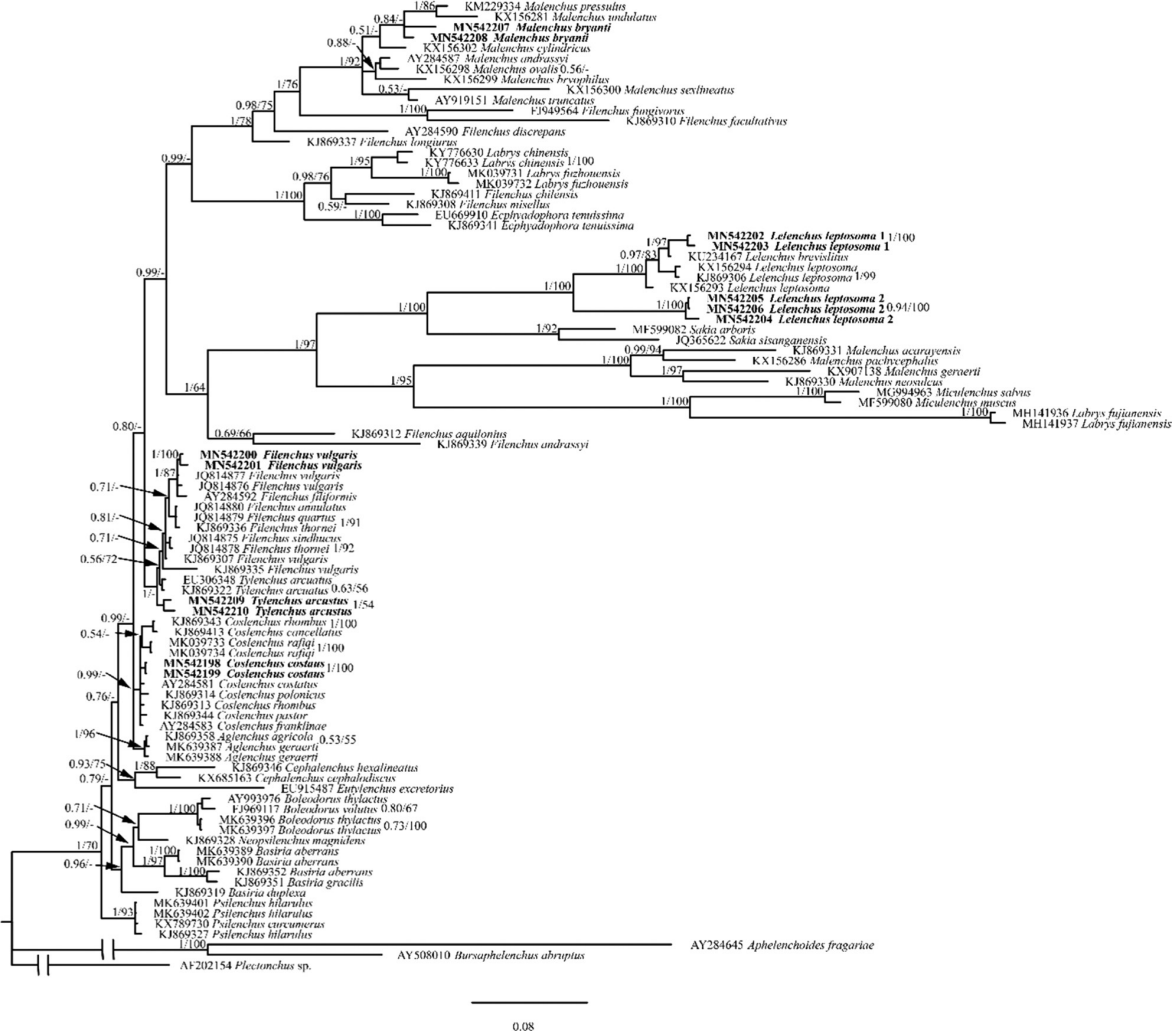
Bayesian 50% majority rule consensus tree interfered with the 18S rRNA gene. New sequences original to this study are indicated in bold. Branch support is indicated in the following order: PP value in BI analysis/BS value from ML analysis.

**Figure 7:**
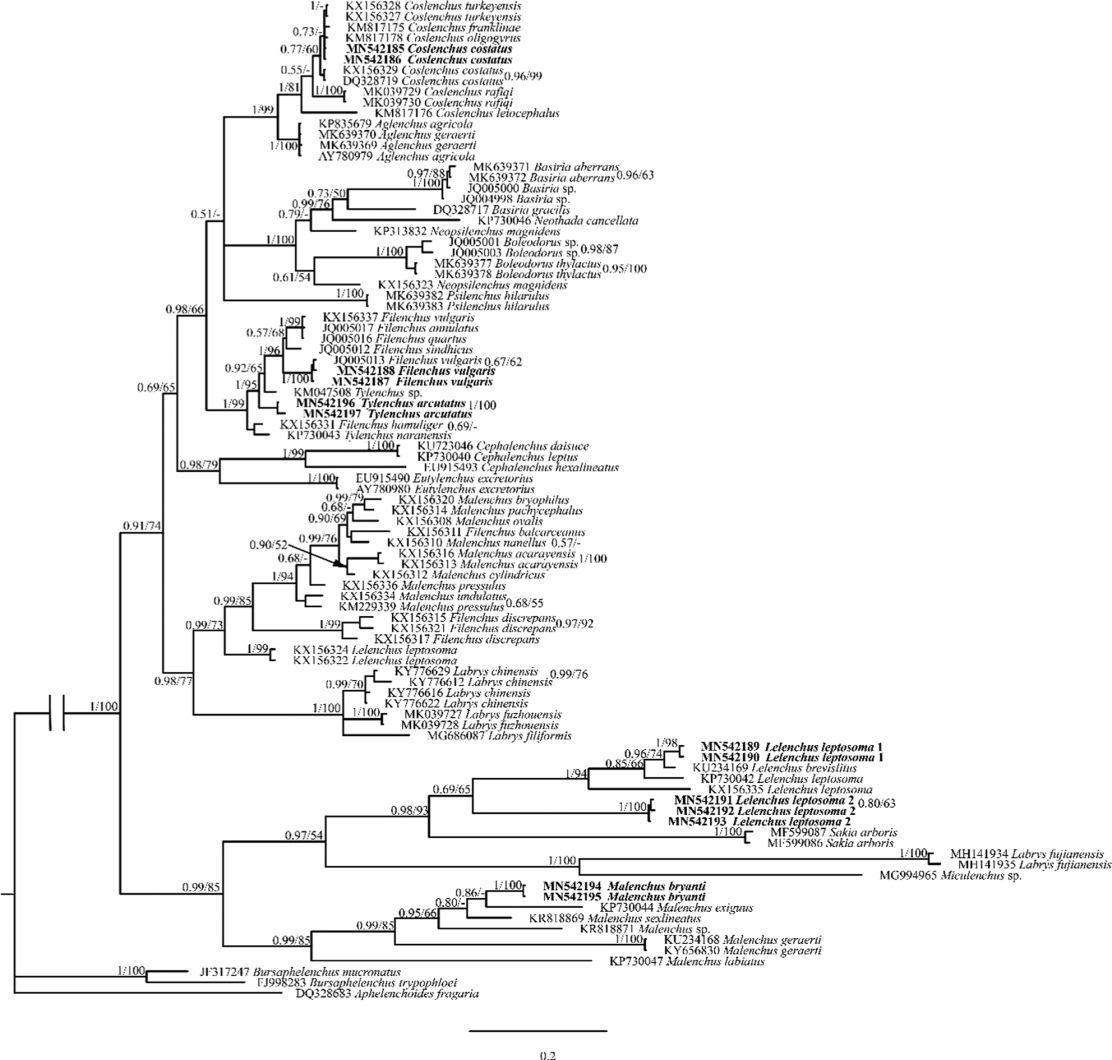
Bayesian 50% majority rule consensus tree interfered with the 28S rRNA gene. New sequences original to this study are indicated in bold. Branch support is indicated in the following order: PP value in BI analysis/BS value from ML analysis.

Although COI phylogeny was unable to reject rRNA phylogenies with full confidence, several COI placements were incongruent with rRNA phylogenies with moderate support in ML analyses: (i) *Boleodorus thylactus* (MN577607, MN577608) was not sister to *B. aberrans* (MN577605, MN577606); (ii) genus *Coslenchus* is not monophyletic, with *Coslenchus rafiqi* (MN577601, MN577602) more close to *Aglenchus geraerti* (MN577603, MN577604) while *Coslenchus costatus* (MN577611, MN577612) was placed more divergently; (iii) genus *Lelenchus* is not monophyletic; (iv) *Tylenchus arcuatus* (MN577620, MN577621) is not sister to *Filenchus vulgaris* (MN577613, MN577614); (v) *Labrys fuzhouensis* (MN577599, MN577600) is closer to two populations of *L. leptosoma* (MN577595, MN577596, MN577615, MN577616) than to *Labrys fujianensis* (MN577697, MN577698); (vi) two *L. leptosoma* populations were clustered together not as sister of *Lelenchus brevislitus*; (vii) *Psilenchus hilarulus* (MN577609, MN577610) or *B. thylactus* was placed as outgroup of all other Tylenchidae species. Aside from Tylenchidae, species from other taxa were in general agreement with rRNA-based phylogeny (Figs. 6, 7).

## Discussion

In the present study, we recovered two populations of *L. leptosoma* that similar in morphology but divergent in phylogenetic placements. Such inconsistency is not surprising as similar cases have been reported in genus *Malenchus* and *Labrys* (Qing et al., 2017, 2018). *Lelenchus leptosoma* is the most frequently encountered species in the genus *Lelenchus* that includes all *Lelenchus* spp. without distinct incisures. This species shows great variations in morphology, e.g., body length ranges from 470 to 780 μm, tail 145 to 278 μm (Geraert, 2008). We demonstrated that even only with minor morphological variations, two populations can be significantly divergent in genetics. We concur with De Ley (2000) that the extremely small size masks the actual morphological difference in nematodes. Indeed, only a few morphological characters (including SEM) are practically helpful for Tylenchidae diagnosis, and a substantial amount of cryptic species were therefore ignored (Qing and Bert, 2019). Similarly, our two recovered *L. leptosoma* were likely to contain at least one cryptic species. However current knowledge in *Lelenchus* is far from sufficient, especially the type of material and molecular data from different reported populations. Consequently, we followed the suggestion given by De Ley (2000) that the key priority for a difficult taxonomic group is to understand major patterns and clades rather than the compilation of a single taxonomic unit.

The mitochondrial COI gene is one of the most important standard barcoding genes that has been used for almost all animals (Hebert et al., 2004). Its higher mutation rate provides a better differentiation of closely related species and is particularly useful for the identification and description of hybrid or cryptic species (Palomares-Rius et al., 2014; Powers, 2004; Shaw et al., 2013). Although it has only been explored for a limited number of nematode species compared to rRNA (Palomares-Rius et al., 2014), the COI gene has recently received increasing attention for nematode barcoding and phylogeny. In plant-parasitic species, COI data were already available for several important taxa, e.g. *Bursaphelenchus* spp. (Kanzakiand and Giblin-Davis, 2012; Ye et al., 2007), *Aphelenchoides* spp. (Sánchez-Monge et al., 2017; Xu et al., XXXX), *Meloidogyne* spp. (Kiewnick et al., 2014), *Pratylenchus* spp. (Janssen et al., 2017; Qing et al., 2019a), and *Scutellonema* spp. (Van den Berg et al., 2013). However, due to the problematic taxonomic status and a lack of taxonomic attention to the Tylenchidae (Qing and Bert, 2019), COI data are only available for one species (*L. brevislitus*) (Soleymanzadeh et al., 2016) regardless of its great diversity. Here we added 27 new COI sequences covering 13 species of Tylenchidae. Our result suggested that the overall resolution of COI phylogeny was low and inferred tree topologies failed to reject rRNA phylogenies. Therefore, we demonstrated that apart from less informative 18S and 28S genes (Qing and Bert, 2019; Qing et al., 2017), COI is also inadequate to resolve Tylenchidae, and therefore searching for valid alternative genes is the key to Tylenchidae phylogeny. Although failing to definitively resolve phylogenies, our analysis of inter-specific/generic differences confirms the validity of COI as a barcode for Tylenchidae. Alongside with our high success rate in PCR amplification using universal COI primer pair JB3/JB4.5 (Bowles et al., 1992), we, therefore, acknowledge the COI as suitable options for Tylenchidae diagnosis.

## 


