# Supplementary material for: Mitochondrial COI gene is valid to delimitate Tylenchidae (Nematoda: Tylenchomorpha) species
Source: J Nematol. 2020 Apr 24;52:e2020-38. doi: 10.21307/jofnem-2020-038 (PMC7266042; doi:10.21307/jofnem-2020-038)
Supplement: Supplementary file 1 [file jofnem-52-038_Supplementary_material.pdf]

SUPPLEMENTARY LEGENDS FOR FIGURES

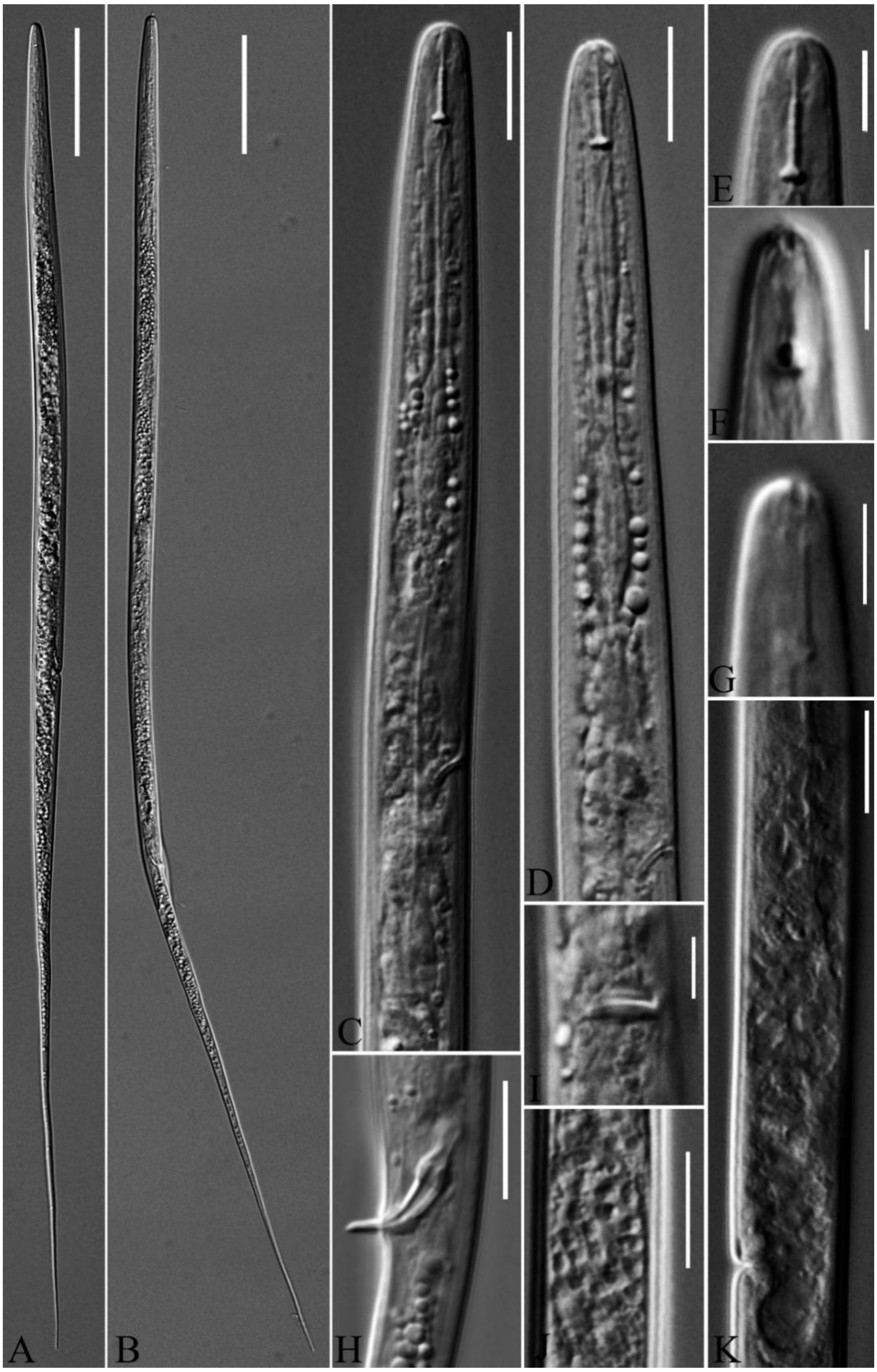

Fig. S1. LM pictures of *Labrys fujianensis*. A, B: Body habitus; C, D: Anterior body; E-G: Different image planes of cephalic region; H: Spicule and gubernaculum; I: Ventral view of vulva; J: Spermatheca; K: Lateral view of female reproductive system. (Scale bar: A, B = 50 μm; C, D, H, J, K = 10 μm; E-G, I = 5 μm.)

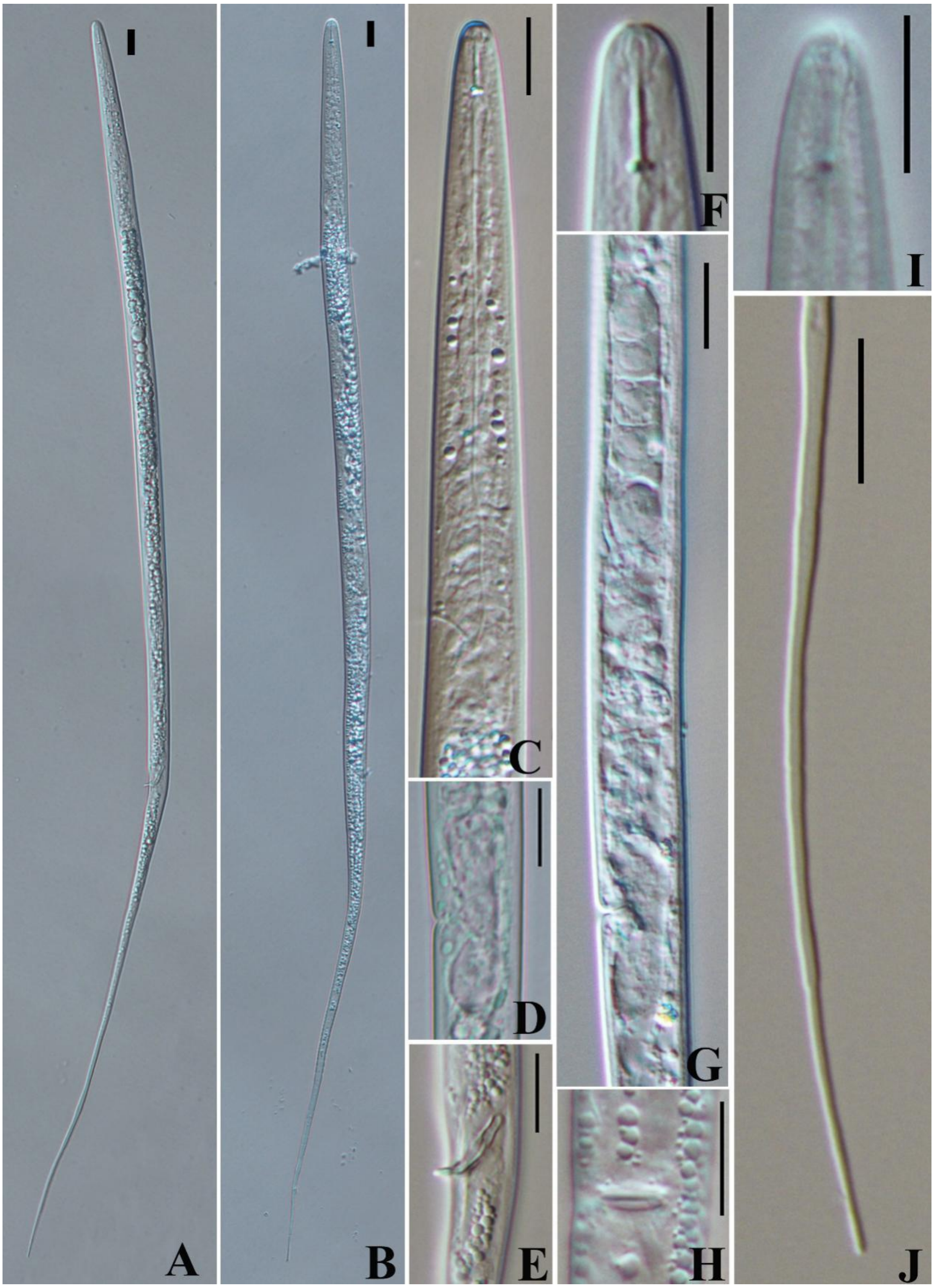

Fig. S2. LM pictures of *Labrys fuzhouensis*. A, B: Body habitus; C: Anterior body; D, G: Lateral view of female reproductive system; E: Spicule and gubernaculum; F, I: Different image planes of cephalic region; H: Ventral view of vulva; J: Tail. (Scale bars = 10 μm.)

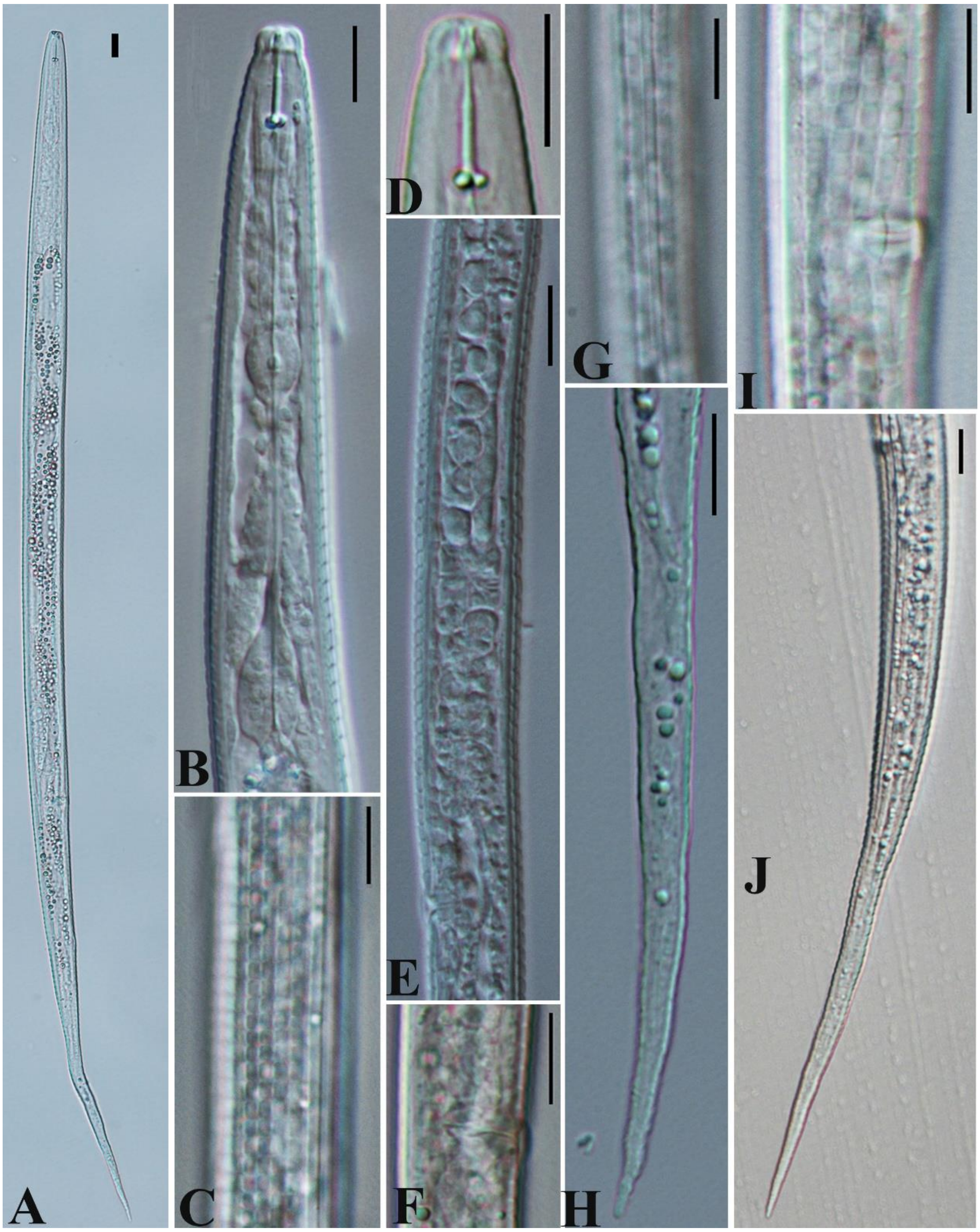

Fig. S3. LM pictures of *Coslenchus rafiqi* (Siddiqui & Khan, 1983) Siddiqi, 1986. A: Female body; B: Anterior body; C, G, I: Longitudinal ridges and incisures; D: Cephalic region; E: Lateral view of female reproductive system; F: Lateral view of vulva; H, J: Tail. (Scale bar = 10 μm.)

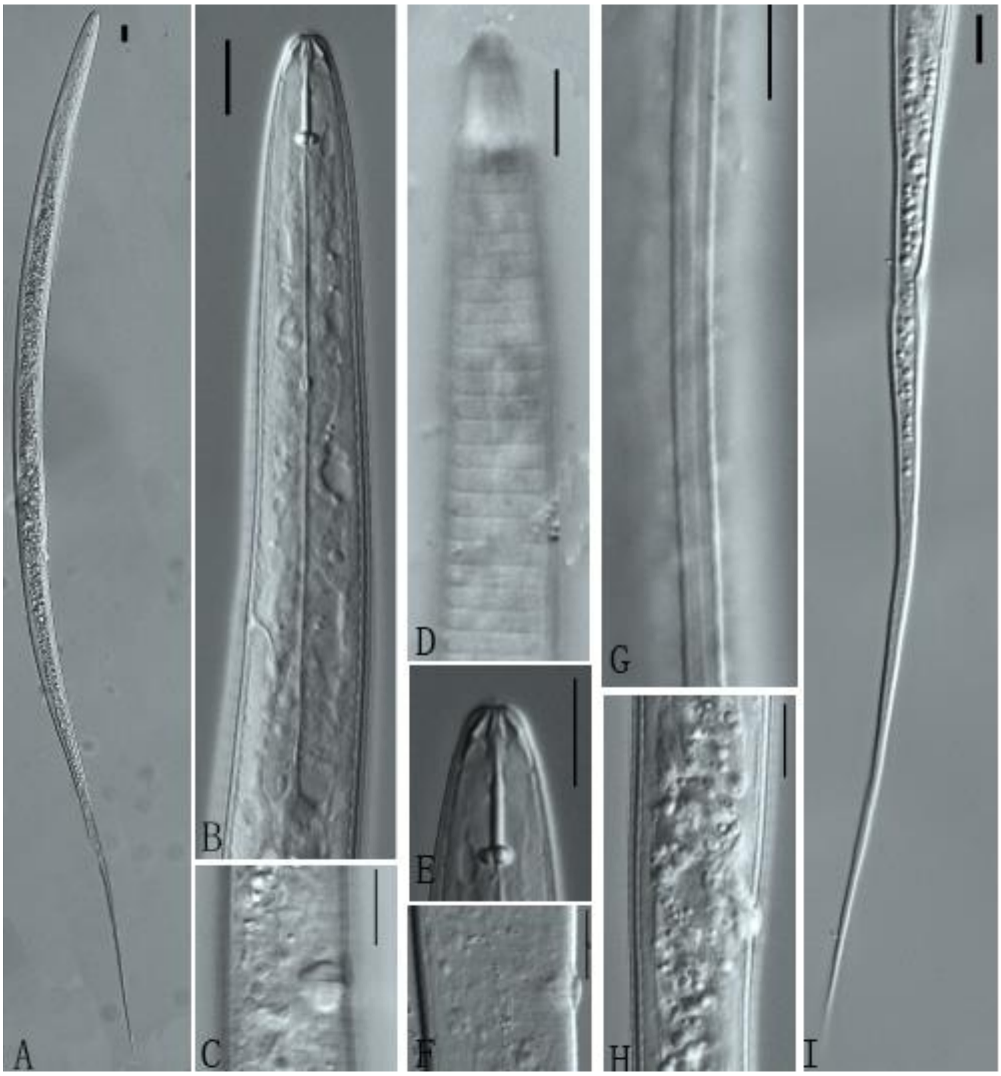

Fig. S4. LM pictures of female of *Aglenchus geraerti*. A：Female habitus; B: Anterior body; C, E: Leteral vulval; D: Annulation; F: Cephalic region; G: Longitudinal lines; H: Tail. (Scale bars=10 μm)

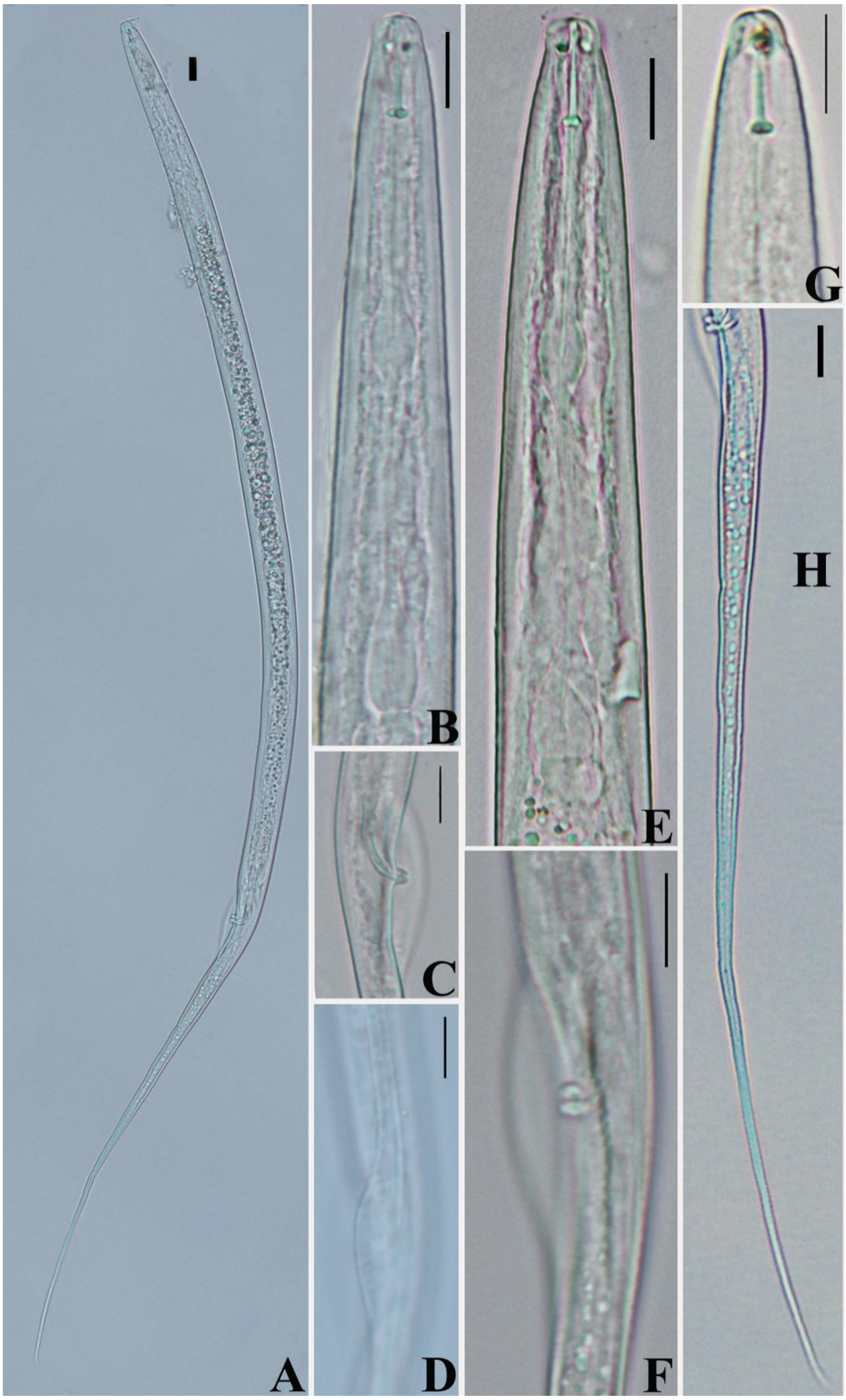

Fig. S5. LM pictures of male of *Aglenchus geraerti*. A：Male habitus; B,E: Anterior body; C,D,F：Spicule and gubernaculum and papilla; G: Cephalic region; H: Cloacal aperture and Tail. (Scale bars=10 μm).

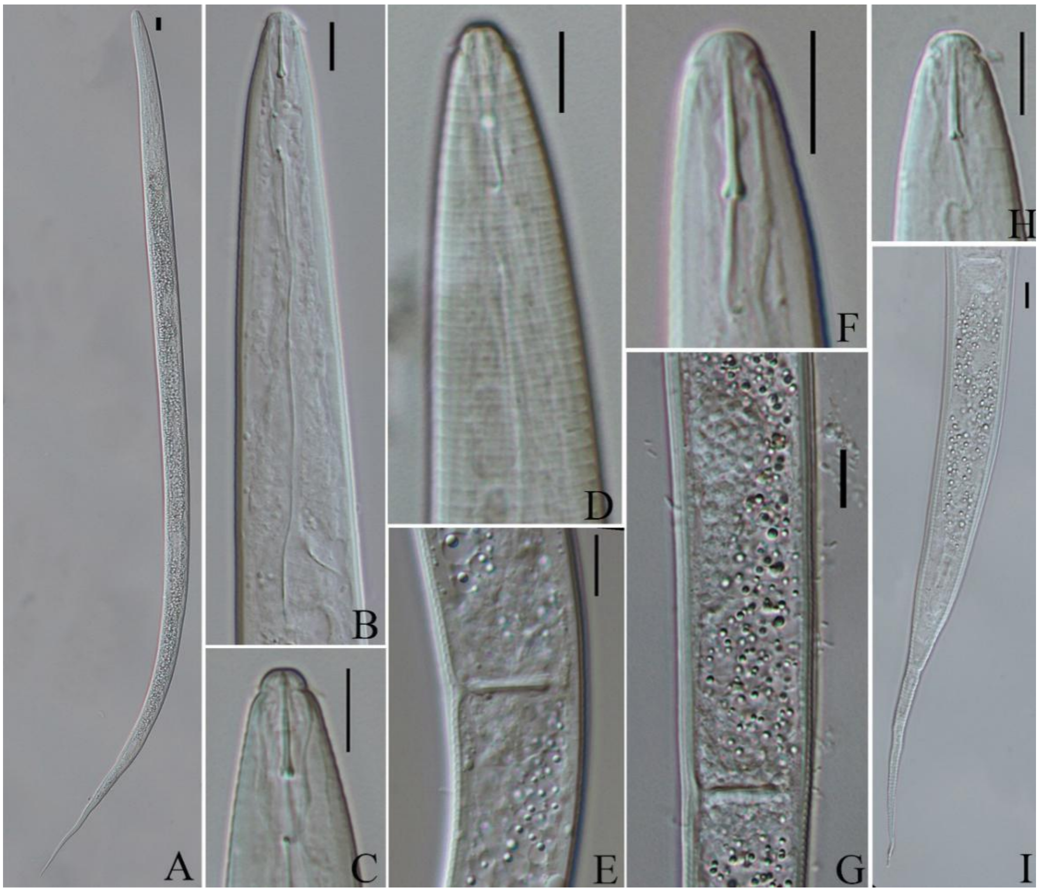

Fig. S6. LM pictures of female of *Basiria aberrans.* A：Female habitus; B: Anterior body; C、F、H: Cephalic region; D: Annulation; E、G: Vulva; I: Tail. (Scale bars=10 μm)

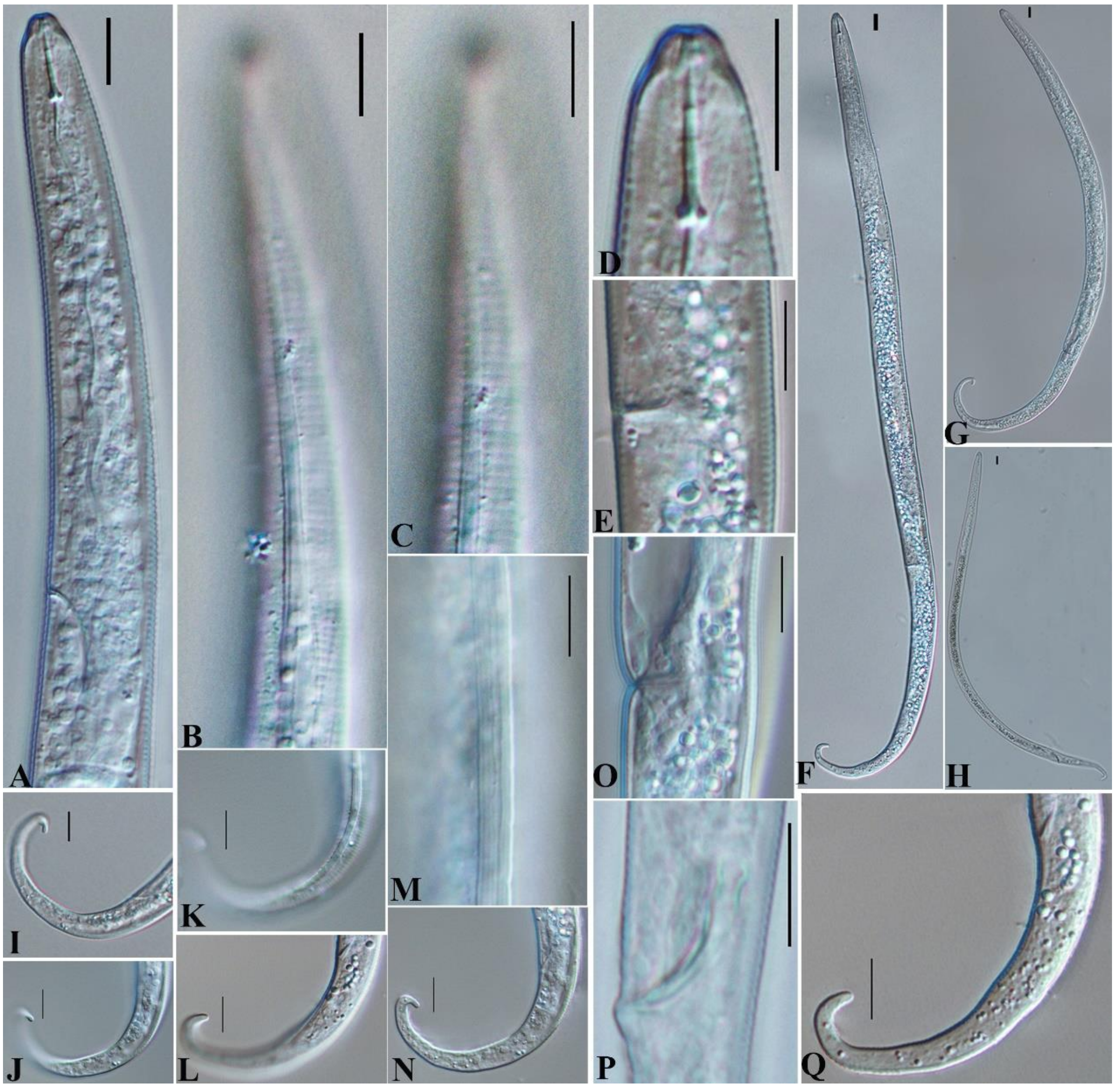

Fig. S7 LM pictures of *Boleodorus thylactus*. A： Anterior body ; B,C,M: Lateral line; D: Cephalic region; E,O: Vulva; F,G: Female habitus; H: Male habitus; I,J,K,L,M,Q: Tail; P: Spicules.(Scale bars=10 μm)

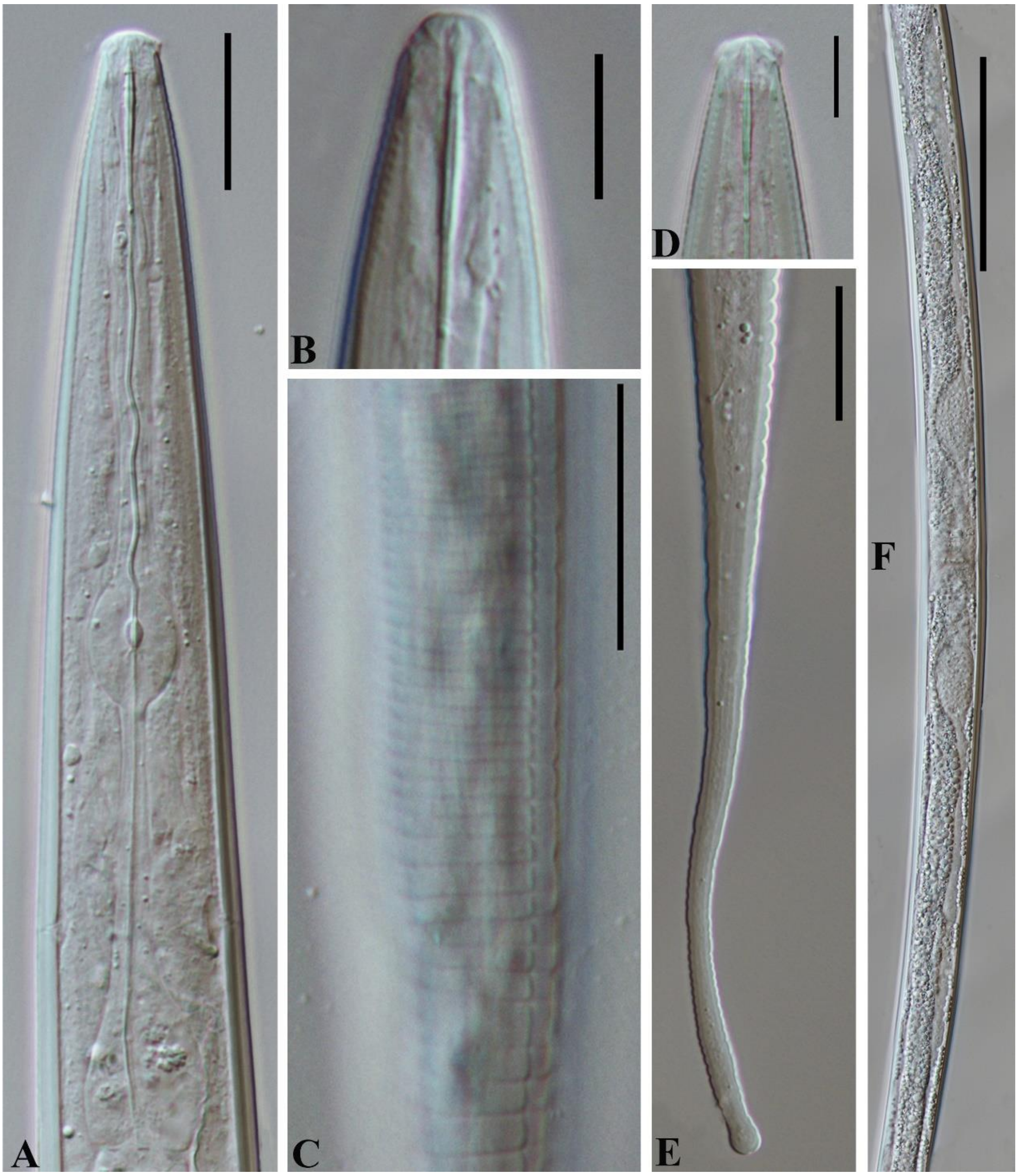

Fig. S8. LM pictures of female of *Psilenchus hilarulus*. A: Anterior body; B,D: Cephalic region; C: Annulation; E: Tail; F: Female reproductive system. (Scale bar = 10 μm)

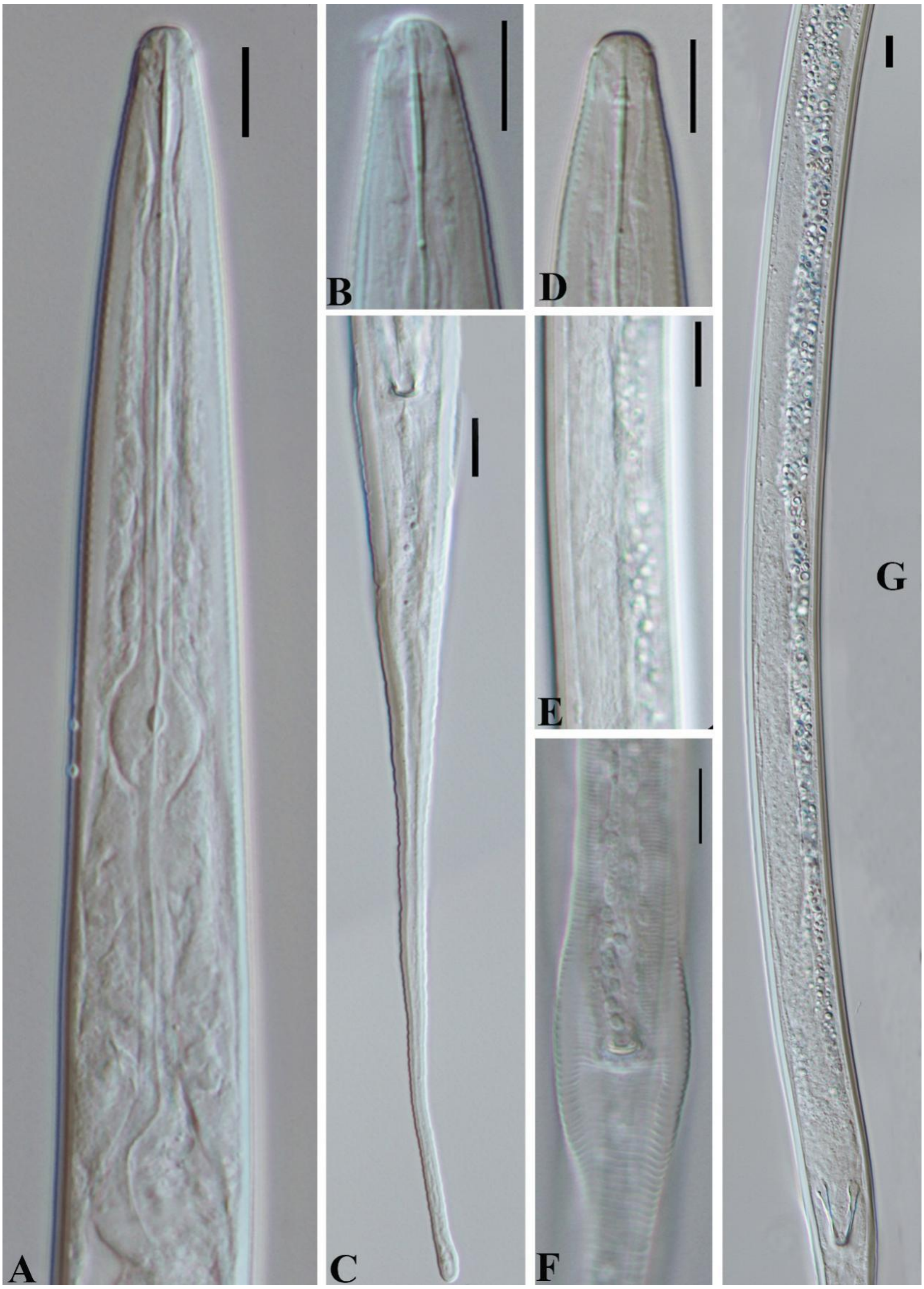

Fig. S9. LM pictures of male of *Psilenchus hilarulus*. A: Anterior body; B,D：Cephalic region C: Tail; E: Lateral field; F: Annulation; G: Reproductive system. (Scale bar = 10 μm)

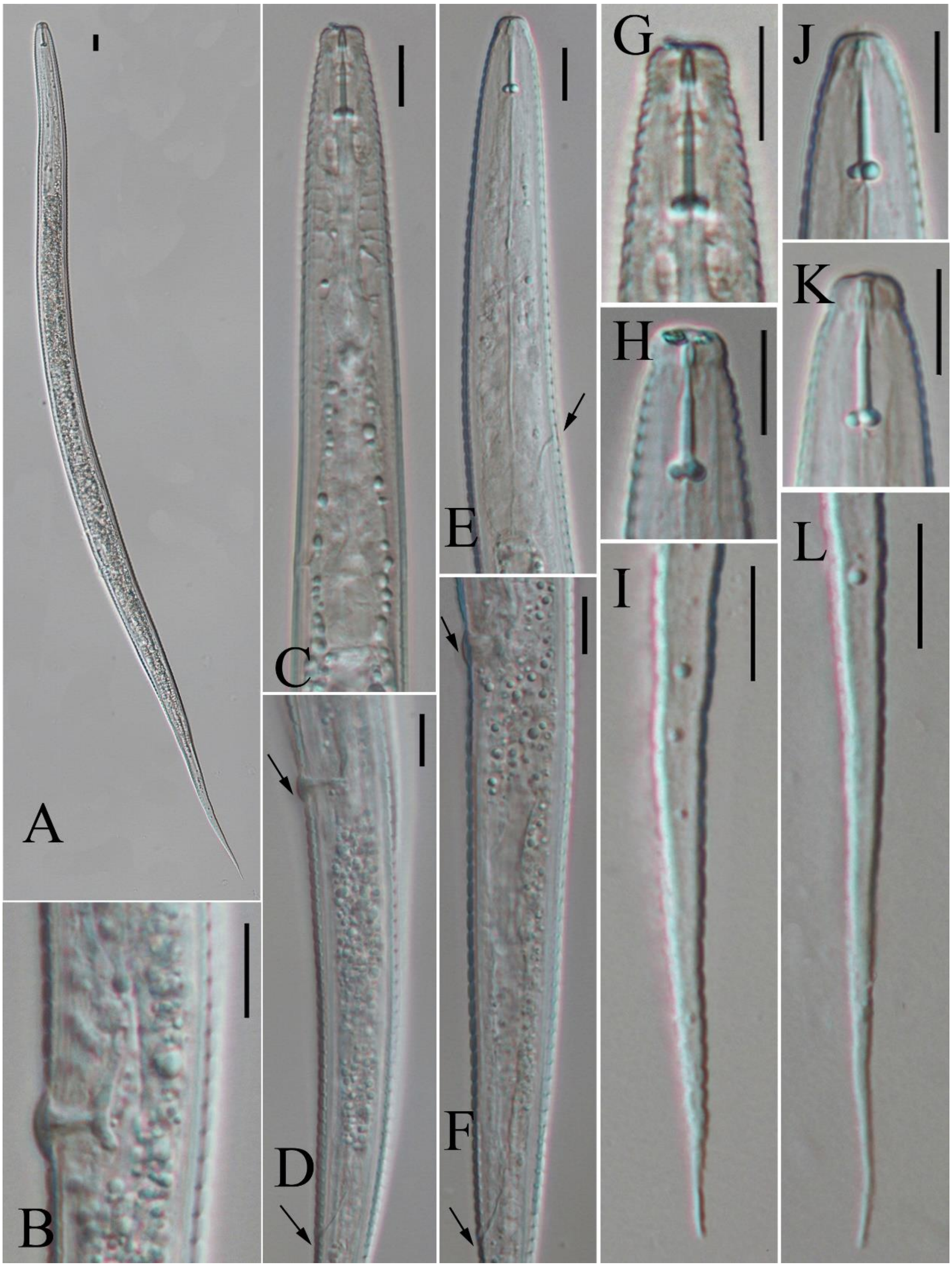

Fig. S10. LM pictures of *Coslenchus costatus*. A: Female habitus; B: Vulva; C,E: Anterior body; D,F: Vulval to anus; G,H,J,K： Cephalic region; I, L: Tail. (Scale bar = 10 μm)

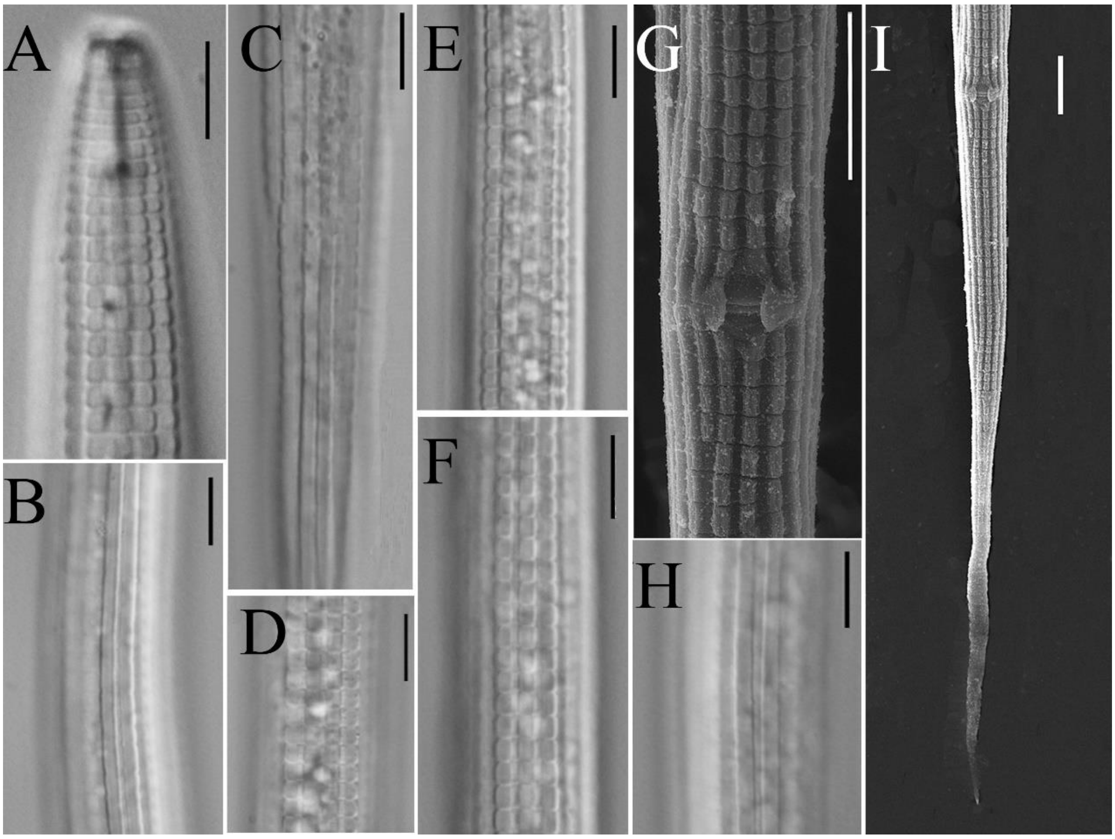

Fig. S11. LM and SEM pictures of *Coslenchus costatus* longitudinal ridges and incisures.

A: Anterior body; B, H: Lateral field; C-F: Longitudinal ridges and incisures; G: Vulval; I: Tail. (Scale bar = 10 μm)

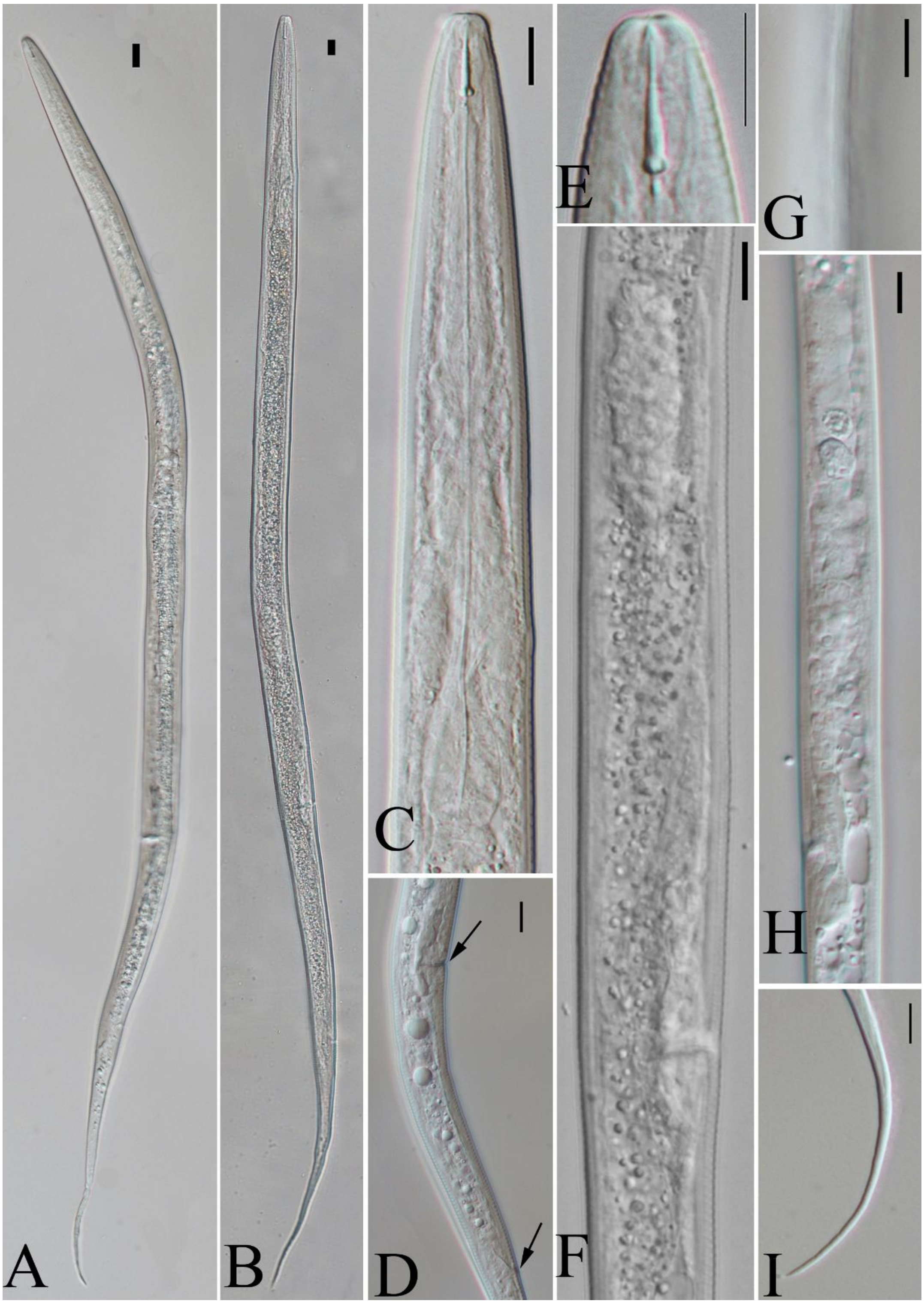

Fig. S12. LM pictures of female of *Filenchus vulgaris.* A, B： Female habitus; C: Anterior body; D: Vulval to anus; E: Cephalic region; F, H: Lateral view of female reproductive system; G: Longitudinal lines; I: Tail. (Scale bars=10 μm)

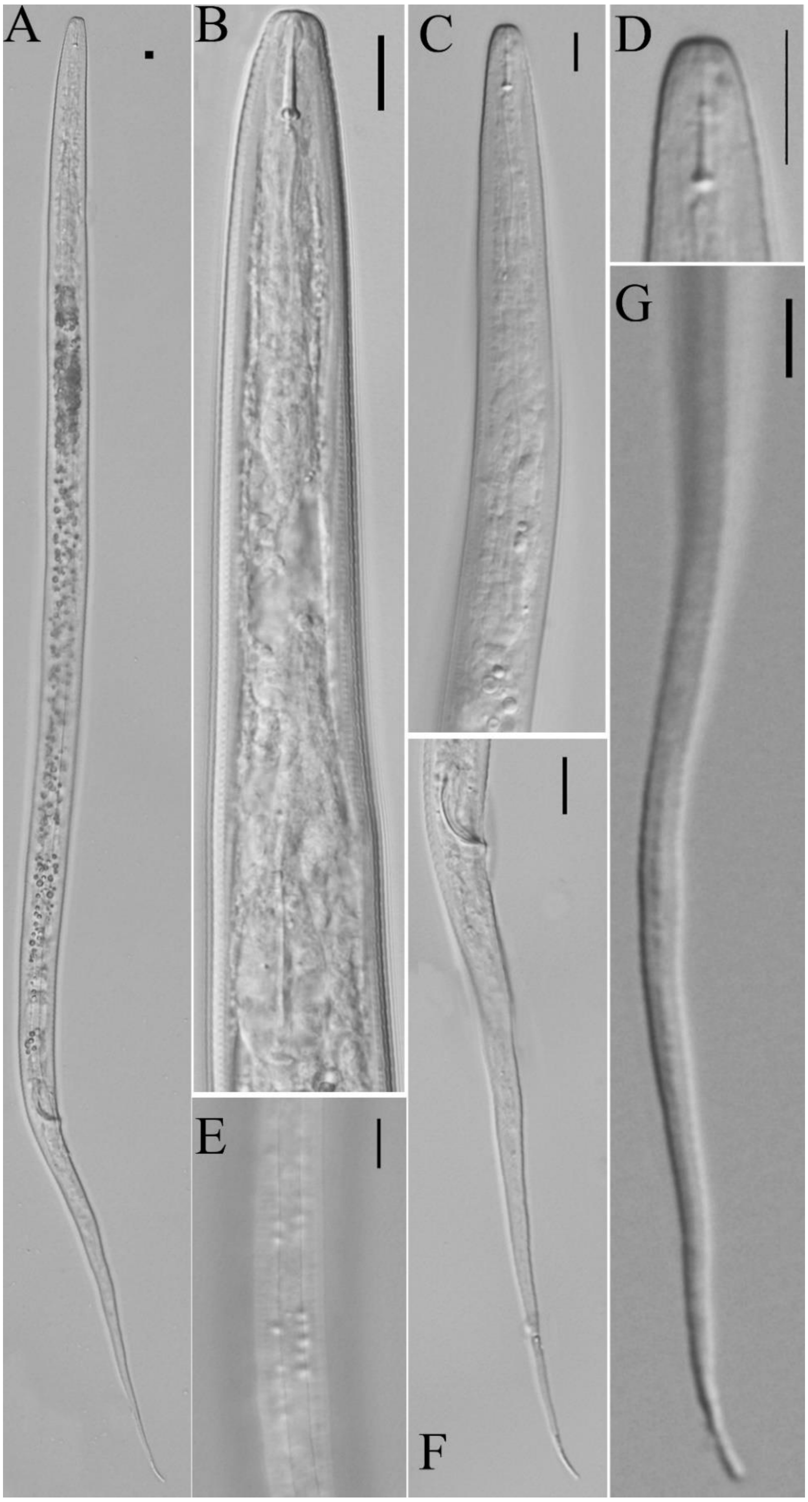

Fig. S13. LM pictures of male of *Filenchus vulgaris*.A：Male habitus; B,C : Anterior body; D：Cephalic region; E: Longitudinal lines: Cloacal aperture and Tail; G: Tail. (Scale bar = 10 μm)

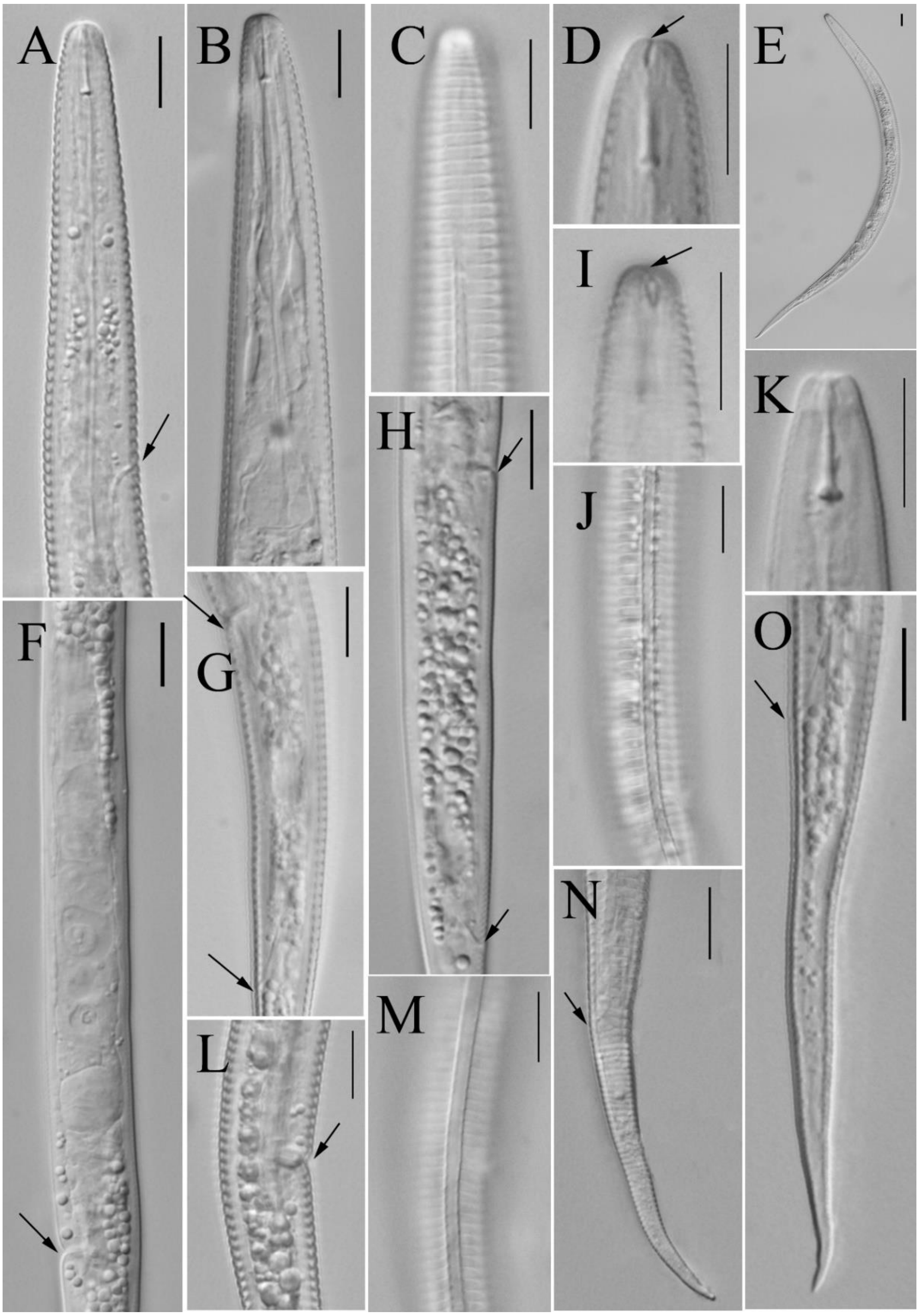

Fig. S14. LM pictures of *Malenchus bryanti* A, B: Anterior body; C: Annulation; D, I, K: Different image planes of cephalic region; E: Body habitus; F: Lateral view of female reproductive system; G, H: Vulval to anus; J, M: Longitudinal lines; L: Vulva; N,O: Tail. (Scale bar = 10 µm)

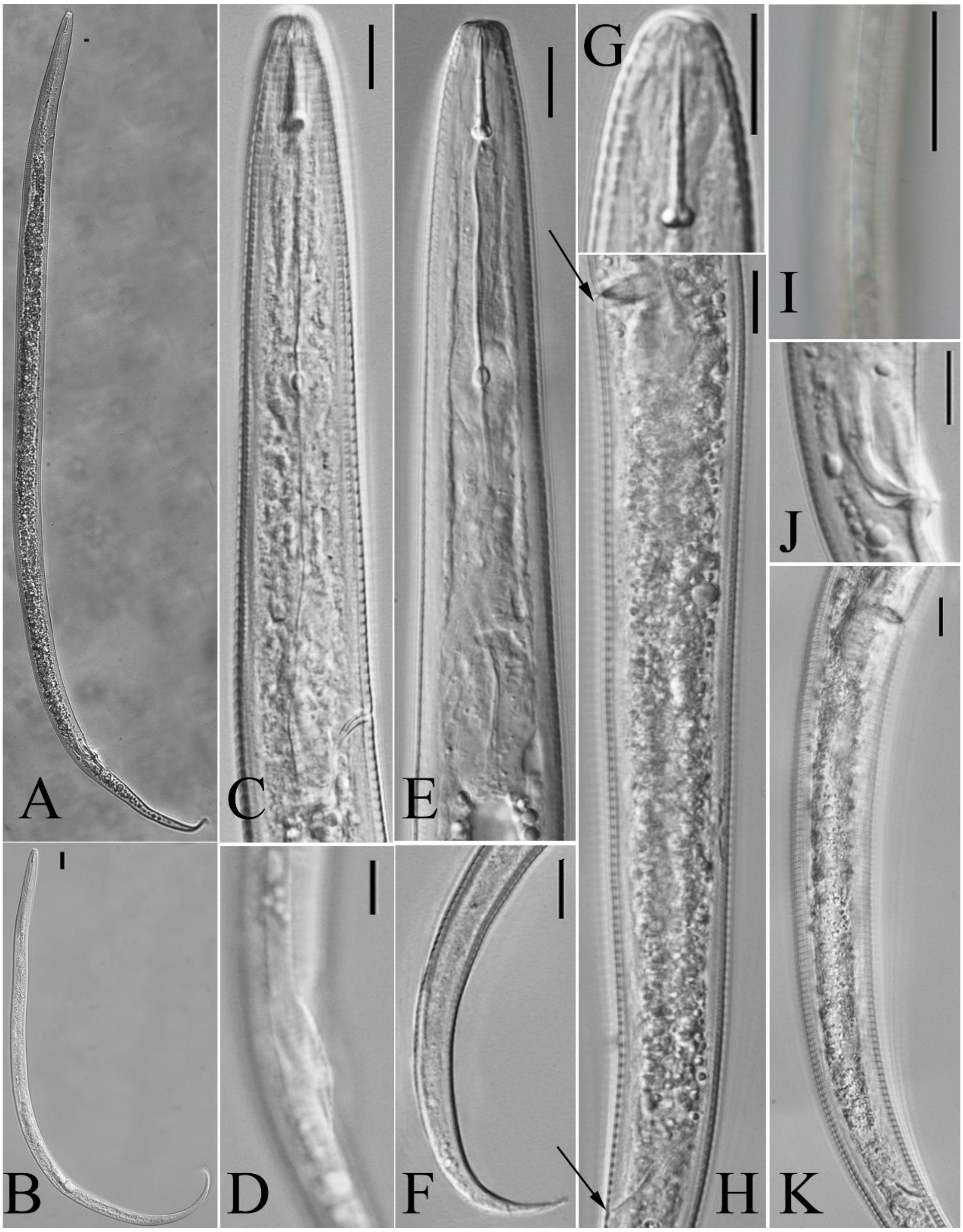

Fig. S15. LM pictures of *Tylenchus arcuatus*. A, B: Body habitus; C, E: Anterior body; D, J: Spicule and gubernaculum; F: Tail; G: Cephalic region; H, K: Vulval to anus; I: Lateral field. (Scale bar = 10 μm)

SUPPLEMENTARY TABLES

Table S1. Morphometric data for *Labrys fuzhouensis*, *Labrys fujianensis*, *Coslenchus rafiqi*. All measurements are in μm and in the form: mean ± s.d. (range).

Character	*Labrys fujianensis*		*Labrys fuzhouensis*		*Coslenchus rafiqi*
	Female	Male	Female	Male	Female
n	16	13	9	7	6
L	563 ±22 （524-599）	552 ±20 （525-589）	524 ±20 （495-564）	526 ±20 （498-559）	498±13.3 （478-518）
L’	354 ±19 （331-379）	-	337 ±10.6 （327-354）	-	391±5.3 （385-398）
a	47.5 ±4.9 （41.3-61）	51.9 ±4.78 （42.8-60）	46.7 ±4.4 （40.6-53.1）	49.2 ±5.0 （41.8-55.4）	29.5±2.7 （24.9-32.8）
b	6.2 ±0.3 （5.6-6.8）	6.1 ±0.17 （5.8-6.3）	6.1 ±0.2 （5.9-6.5）	5.8 ±0.2 （5.6-6.1）	5.3±0.4 （4.5-5.6）
c	2.8 ±0.2 （2.4-3.0）	2.7 ±0.18 （2.5-3.2）	2.9 ±0.1 （2.8-3.0）	2.7 ±0.2 （2.5-2.9）	4.6±0.3 （4.3-4.9）
c’	26.5 ±2.6 （21.3-29.5）	25.5 ±3.2 （20.3-29.8）	25.0 ±1.7 （22.6-26.4）	25.0 ±2.5 （22.4-28.4）	11.2±0.8 （10.4-12.1）
V or T	49.8 ±2.8 （45.1-56.5）	23.9 ±1.3 (21.8-25.0)	50.9 ±1.8 （48.7-53.9）	-	64.2±1.0 （62.9-66.0）
V’	78.7 ±1.1 （76.7-80.3）	-	79.0 ±1.3 （77.2 -80.0）	-	81.6±2.8 （79.0-84.6）
Stylet	8.9 ±0.7 （7.6-10.0）	8.3 ±0.6 (7.2-9.2)	7.2 ±0.7 （6.0-8.2）	7.3 ±0.3 （6.9-7.8）	9.75±0.26 （9.4-10.1）
MB	50 ±3.4 （45.2-55）	43.6 ±3.4 (39-48.5)	49.5 ±3.7 （42.5-52.8）	52.6 ±4.1 (48.0-55.8)	52.3±3.5 （47.0-56.3）
Excretory pore to anterior end	66 ±3.5 (60-71)	65 ±3.5 (60-70)	64.4 ±1.3 （62.4-65.6）	65.5 ±2.4 （61.5-68.7）	76.7±3.5 （71.6-81.2）
Head-vulva	279 ±12.3 （261-302）	-	267 ±9 （255-283）	337 ±23 （305-379）	320±7.7 （308-330.9）
Vulva-anus	75 ±5.2 （69-86）	-	70. 1±2.9 （65.7-73.7）	-	71.7±7.8 （61.1-79.5）
Body width	12.0 ±1.2 （9.6-13.6）	10.7 ±0.9 (9.3-12.4)	11.3 ±0.9 （10.1-12.6）	10.8 ±1.2 （9.4-12.8）	17.0±1.3 （15.3-19.2）
Tail	204 ±13.7 (184-225)	209 ±16.3 (165-227)	176 ±4 （172-182）	193 ±7 (178-202)	109±7.8 （100-119）
Anus width/Cloacal width	7.8 ±0.9 （6.6-9.3）	8.3 ±0.8 （7.2-10.0）	7.1 ±0.3 （6.8-7.6）	7.8 ±0.7 （6.7-8.8）	9.8±1.2 （8.9-11.4）
Vulva width	10.4 ±0.6 （9.3-11.6）	-	10.0 ±0.7 （8.7-11.3）	-	1.6±0.4 （1.3-2.0）
Post-uterine sac	10.5 ±1.7	-	11.5 ±0.6	-	-

	（8.1-13.4）		（10.8-12.4）		
Tail/ Vulva to anus	-	-	-	-	1.6 ±0.4 （1.3-2.0）
Spicules	-	12.2 ±1.0 (10.8-13.6)	-	11.3 ±0.6 （10.3-12.1）	-
Bursa	-	21 ±1.7 (19.1-23)	-	16.1 ±1.5 （14.7-18.6）	-

Table 2. Morphometric data for *Boleodorus thylactus*, *Aglenchus geraerti*, *Coslenchus costatus*. All measurements are in μm and in the form: mean ±s.d. (range).

Character	*Boleodorus thylactus*		*Aglenchus geraerti*		*Coslenchus costatus*
	Female	Male	Female	Male	Female
n	10	4	6	4	6
L	523±39.8 (460-582)	499±34.6 (463-551)	632±57 (546-723)	560±32.7 (514-606)	555±17.3 (525-583)
L'	450±35.6 (395-505)	-	389.9±33.3 (356-423)	-	447±17.7 (426-469)
a	26.2±2.9 (21.3-30.9)	34.6±3.5 (29.4-36.9)	31.7±4.5 (27.3-35.6)	36.5±3.2 (33.5-39.6)	27.1±1.7 (25.4-30.0)
b	4.8±0.20 (4.6-5.2)	4.3±0.05 (4.2-4.3)	6.3±0.9 (5.1-7.6)	6.0±0.34 (5.7-6.5)	5.3±0.10 (5.1-5.4)
c	7.2±0.30 (6.7-7.6)	6.6±0.22 (6.3-6.9)	3.0±0.02 (3.01-3.04)	2.9±0.82 (2.9-3.0)	4.9±0.56 (4.3-5.2)
c'	6.7±0.32 (6.0-7.2)	7.0±0.57 (6.4-7.6)	18.9±1.19 (18.0-19.7)	-	11.6±1.5 (9.9-12.5)
V or T	65.6±1.5 (63.0-68.8)	-	54.1±4.2 (50.1-60.6)	-	63.4±0.91 (61.8-64.1)
V'	76.2±1.5 (73.4-78.0)	-	82.5±6.7 (77.8-87.2)	-	80.6±2.6 (79.0-83.6)
Stylet	9.3±0.69 (8.2-10.2)	9.3±0.56 (8.6-10.1)	11.5±0.70 (10.2-12.3)	9.5±0.99 (8.4-10.8)	11.6±0.21 (11.4-12.0)
MB	55.3±1.7 (53.6-57.0)	52.5	53.4±0.80 (52.4-54.6)	49.3±1.6 (47.8-51.1)	50.3±1.5 (48.8-53.2)
Excretory pore to anterior end	88.7±5.4 (79.6-96.3)	88.9±3.6 (83.9-92.8)	82.1±4.4 (78.4-88.4)	70.7±1.3 (68.8-72.0)	81.6±5.7 (72.5-87.8)
Head-vulva	343±28.3 (290-384)	423±30.7 (393-469)	341±20.1 (311-368)	-	351±13.1 (334-371)
Vulva-anus	107.5±9.5 (91.2-122)	-	95.7±2.7 (93.0-98.4)	-	94.1±5.6 (88.5-103)
Body width	20.2±2.6 (15.7-24.2)	14.5±1.1 (12.8-15.8)	19.6±1.6 (17.9-22.3)	15.4±0.70 (14.6-16.5)	20.5±1.3 (18.3-21.8)
Tail	72.4±5.7 (64.3-80.1)	75.6±3.1 (72.6-80.4)	195.3±16.0 (179-211)	192±12.5 (179-212)	116±11.0 (105-131)
Anus width/Cloacal width	10.9±1.1 (8.9-12.2)	10.9±0.50 (10.0-11.4)	16.7±2.1 (14.8-20.1)	10.4±0.74 (9.9-11.4)	10.2±2.2 (8.4-13.2)
Vulva width	17.4±1.4 (15.3-19.5)	-	-	-	17.8±1.3 (18.3-21.8)
Post-uterine sac	7.8±1.8 (5.5-9.9)	-	-	-	-
Tail/ Vulva to anus	0.68±0.04	-	2.0±0.15	-	1.2±0.21

	（0.61-0.74）		（1.9-2.2）		（1.1-1.5）
Spicules	-	14.7±0.57<br>（14.3-15.7）	-	14.9±1.2<br>(13.7-16.5)	-
Bursa	-	13.2	-	27.3±3.3<br>(23.6-31.2)	-

Table 3. Morphometric data for *Basiria aberrans*, *Filenchus vulgaris*, *Lelenchus leptosome* 2, *Lelenchus leptosome* 1. All measurements are in μm and in the form: mean ±s.d. (range).

Character	*Basiria aberrans*	*Filenchus vulgaris*		*Lelenchus leptosome* 2		*Lelenchus leptosome* 1
	Female	Female	Male	Female	Male	Female
n	10	9	3	15	10	7
L	727±56.4 (665-845)	736±114 (503-862)	873±72.9 (782-960)	450 ±26.3 (423-525)	438±17.1 (413-477)	511±20.1 (473-528)
L'	586±28.1 (564-626)	640±10.4 (626-649)	-	-	-	-
a	31.1±3.5 (26.8-35.4)	28.6±3.1 (24.1-33.2)	33.8±4.6 (30.2-38.9)	37.2 ±2.9 (31.0-42.0)	42.4±3.5 (36.8-47.0)	42.1±3.1 (37.4-46.1)
b	6.1±0.43 (5.7-6.7)	5.8±0.45 (5.2-6.6)	1.3±0.02 (1.3-1.3)	5.3 ±0.32 (4.7-5.9)	5.1±0.24 (4.7-5.5)	4.7±0.69 (3.9-5.7)
c	6.7±0.21 (6.5-6.9)	5.1±0.10 (5.0-5.2)	4.01±0.45 (3.49-4.30)	3.2 ±0.18 (2.8-3.5)	3.1±0.19 (2.8-3.3)	3.7±0.63 (3.2-4.4)
c'	11.1±0.96 (10.4-12.2)	10.5±1.1 (9.3-11.4)	10.2±1.7 (9.1-12.1)	19.7 ±1.2 (17.6-21.2)	20.5±2.9 (16.4-24.1)	18.8±3.3 (15.3-21.9)
V or T	65.3±1.6 (63.5-67.9)	62.2±2.4 (57.3-64.5)	-	54.0 ±1.6 (50.6-56.3)	22.7±2.1 (20.4-24.5)	54.7±2.2 (52.3-58.9)
V'	76.4±0.4 (75.9-76.6)	80.6±3.3 (78.5-84.4)	-	78.1 ±1.3 (75.8-79.3)	-	78.2±0.44 (77.9-78.7)
Stylet	11.2±0.55 (10.3-12.0)	11.4±2.5 (5.7-14.1)	11.3±2.4 (8.1-13.8)	6.3 ±0.54 (5.6-7.4)	5.9±0.29 (5.5-6.4)	6.9±0.79 (5.8-8.0)
DGO	7.6±0.7 (6.2-8.6)	1.2±0.30 (0.95-1.9)	1.1±0.39 (0.75-1.7)	0.74±0.12 (0.60-0.90)	0.91±0.19 (0.69-1.2)	0.95±0.38 (0.63-1.5)
Excretory pore to anterior end	89.9	99.1±4.6 (94.0-105)	-	62.6±4.6 (57.5-73.8)	61.4±2.9 (58.1-67.2)	76.0±1.0 (74.4-77.0)
Pharynx	105±6.6 (92.9-116)	112±15.5 (82.8-127)	130±15.7 (117-152)	76.5±5.5 (66.0-88.6)	75.8±3.4 (69.0-81.6)	95.1±4.3 (89.5-100)
MB	62.4±2.1 (59.3-65.2)	47.3±9. (38.1-70.1)	39.7±16.3 (24.1-55.8)	47.7 ±3.3 (45.6-57.4)	48.7±1.9 (45.9-50.1)	45.5±3.3 (41.8-49.5)
Head-vulva	-	-	-	242.7±16.0 (220-288)	-	279±4.3 (273-286)
Vulva-anus	141.1±5.7 (135-149)	145±5.1 (140-152)	-	68.1±5.3 (61.1-80.6)	-	77.5±1.3 (75.9-79.0)
Body width	23.7±3.3 (19.0-29.0)	25.8±3.3 (18.8-29.5)	25.9±1.0 (24.7-27.2)	12.2±1.3 (10.6-15.3)	10.4±0.82 (9.3-12.3)	13.2±2.8 (10.9-20.2)
Tail	102±0.97 (101-103.3)	161±8.0 (152-167)	222±39.5 (182-275)	144±11.4 (128-159)	143±7.9 (123-154)	139±24.0 (148-166)
Anus width/Cloacal width	9.3±0.64 (8.4-9.7)	15.5±1.8 (14.1-18.0)	21.7±1.2 (20.0-22.7)	-	-	-

Vulva width	20.9±1.9 （18.5-23.9）	15.7±3.6 （12.4-20.7）	-	10.7±1.02 （9.7-13.8）	-	-
Tail/ Vulva to anus	0.72±0.04 （0.68-0.75）	1.1±0.05 （1.1-1.2）	-	2.1±0.21 （1.9-2.5）	-	-
Spicules	-	-	21.1±2.0 （19.3-23.9）	—	12.5±0.95 （11.2-14.7）	-
Bursa	-	-	42.2±2.3 （39.8-44.5）	-	16.8±2.8 （13.8-21.4）	-

Table 4. Morphometric data for *Malenchus bryanti*, *Tylenchus arcuatus* and *Psilenchus hilarulus*. All measurements are in μm and in the form: mean ± s.d. (range).

Character	*Malenchus bryanti*	*Tylenchus arcuatus*		*Psilenchus hilarulus*	
	Female	Female	Male	Female	Male
n	11	5	1	10	10
L	321 ±24.3 (296-374)	754 ±20.9 (727-780)	746	1011 ±69.7 (925-1141)	847 ±60.2 (731-948)
L'	257 ±3.5 (251-260)	629 ±29.2 (599-667)	-	850	-
a	20.5 ±4.1 (16.2-27.8)	30.3 ±2.5 (27.8-34.1)	29.7	33.4 ±3.0 (29.6-40.0)	39.4 ±2.6 (35.0-43.6)
b	4.4 ±0.29 (4.0-4.9)	6.2 ±0.48 (5.3-6.6)	6.1	6.7 ±0.63 (6.1-7.9)	5.9 ±0.38 (5.1-6.3)
c	6.1 ±0.97 (5.0-7.4)	7.4 ±0.25 (7.1-7.7)	6.2	9.5	5.7 ±0.21 (5.3-6.0)
c'	6.4 ±0.90 (5.4-7.3)	7.7 ±0.60 (7.1-8.3)	6.5	8.0	9.6 ±0.92 (8.1-10.7)
V or T	64.9 ±3.3 (56.0-67.6)	64.6 ±1.4 (63.2-67.3)	13.9	49.9 ±2.4 (47.9-56.2)	38.2 ±3.3 (35.3-44.6)
V'	78.4 ±1.0 (78.2-80.3)	78.0 ±2.3 (76.2-81.4)	-	56.5	-
Stylet	7.1 ±0.59 (6.1-8.3)	14.3 ±0.42 (13.9-15.0)	13.7	13.5 ±1.1 (11.4-14.5)	12.5 ±0.66 (10.9-13.2)
DGO	0.37 ±0.10 (0.22-0.61)	-	-	6.4 ±0.49 (5.2-6.9)	7.1 ±0.62 (5.9-8.0)
Excretory pore to anterior end	61.8 ±3.4 (55.0-65.3)	113.6 ±9.1 (107-129)	-	-	-
Pharynx	62.8 ±2.8 (58.0-67.1)	107 ±11.0 (94.4-127)	104	133.5 ±9.4 (118-153)	127 ±3.9 (120-135)
MB	55.4 ±3.7 (52.4-52.7)	52.0 ±3.8 (45.5-54.7)	38.8	59.7 ±3.5 (55.4-67.6)	61.8 ±1.7 (58.7-64.6)
Head-vulva	208 ±10.4 (192-236)	590 ±17.2 (463-508)	-	504 ±40.8 (455-583)	699 ±56.2 (592-783)
Vulva-anus	52.8 ±4.3 (48.1-59.8)	148 ±9.6 (135-159)	-	353	-
Body width	16.0 ±2.3 (13.3-19.7)	25.0 ±1.3 (22.9-26.5)	25.2	30.4 ±2.0 (27.0-32.7)	21.6 ±1.7 (19.0-24.1)
Tail	51.3 ±7.0 (42. 4-61.4)	107 ±11.0 (94.4-127)	121	95	148 ±7.9 (139-166)
Anus width/Cloacal width	7.7 ±0.59 (6.6-8.4)	13.7 ±1.1 (12.2-14.7)	18.9	11.8	-
Vulva width	13.5 ±1.2 (11.6-15.1)	22.9 ±0.97 (21.6-24.0)	-	29.5 ±2.3 (23.7-32.2)	15.5 ±1.7 (13.1-18.0)

Tail/ Vulva to anus	1.0±0.15<br>（0.88-1.1）	0.74±0.10<br>（0.66-0.85）	-	0.27	-
Spicules	-	-	21.0	-	-
Bursa	-	-	22.8	-	31.5±5.9<br>（24.8-47.0）
